# Geometrically encoded SERS nanobarcodes for the logical detection of nasopharyngeal carcinoma-related progression biomarkers

**DOI:** 10.1038/s41467-021-23789-3

**Published:** 2021-06-08

**Authors:** Duo Lin, Chang-Lin Hsieh, Keng-Chia Hsu, Pei-Hsuan Liao, Sufang Qiu, Tianxun Gong, Ken-Tye Yong, Shangyuan Feng, Kien Voon Kong

**Affiliations:** 1 Key Laboratory of OptoElectronic Science and Technology for Medicine, Ministry of Education, Fujian Provincial Key Laboratory for Photonics Technology, Fujian Normal University, Fuzhou, Fujian China; 2 Department of Chemistry, National Taiwan University, Taipei, Taiwan; 3 Fujian Medical University Cancer Hospital, Fujian Cancer Hospital, Fuzhou, Fujian China; 4 State Key Laboratory of Electronic Thin Films and Integrated Devices, School of Electronic Science and Engineering (National Exemplary School of Microelectronics), University of Electronic Science and Technology of China, Chengdu, China; 5 School of Biomedical Engineering, The University of Sydney, Sydney, NSW Australia; 6 The University of Sydney Nano Institute, The University of Sydney, Sydney, NSW Australia

**Keywords:** Biosensors, Bioanalytical chemistry, Biochemistry

## Abstract

The limited availability of nasopharyngeal carcinoma-related progression biomarker array kits that offer physicians comprehensive information is disadvantageous for monitoring cancer progression. To develop a biomarker array kit, systematic identification and differentiation of a large number of distinct molecular surface-enhanced Raman scattering (SERS) reporters with high spectral temporal resolution is a major challenge. To address this unmet need, we use the chemistry of metal carbonyls to construct a series of unique SERS reporters with the potential to provide logical and highly multiplex information during testing. In this study, we report that geometric control over metal carbonyls on nanotags can produce 14 distinct barcodes that can be decoded unambiguously using commercial Raman spectroscopy. These metal carbonyl nanobarcodes are tested on human blood samples and show strong sensitivity (0.07 ng/mL limit of detection, average CV of 6.1% and >92% degree of recovery) and multiplexing capabilities for MMPs.

## Abstract (web summary)

SERS assays have potential for multiplexed detection of biomarkers but differentiation of SERS tags remains a challenge. Here, the authors report the creation of 14 distinct geometrically controlled metal carbonyl tags and demonstrate multiplexed detection of nasopharyngeal carcinoma biomarkers from patient blood.

## Introduction

Biomarker detection is the most effective strategy to improve the prognosis of patients with nasopharyngeal carcinoma (NPC); therefore, the development of point-of-care test with high sensitivity and specificity for tracking NPC progression is critical for disease management. Raman spectroscopy has been developed as an outstanding vibrational spectroscopy technique for multiplexed molecular analysis^1,2^. This exclusive feature offers the potential for progression evaluation.

Proteases are essential for various pathological functions, such as inflammation and cell invasion^3^. Degradation of the extracellular matrix by matrix metalloproteinases (MMPs) could facilitate tumor invasion^3,4^. MMP-2 and MMP-9 are the two MMPs most closely correlated with metastatic potential due to their collagen cleavage functionality^5^. Numerous MMPs are expressed at higher levels in cancerous tissue than in non-cancerous tissue, and the levels of expression have been revealed to be related to stages of cancer^6^.

Surface-enhanced Raman scattering (SERS)-based biosensors have multiplex capabilities due to fingerprinting Raman spectra and high sensitivity^7–9^. Despite the remarkable success of SERS nanoparticles for multiplexed detection, little effort has been made to create robust barcodes suitable as biosensing probes. Additionally, logical multiplex detection with more than three biomarkers remains challenging^7,10^. Importantly, multiple biomarker detection can allow clinicians to corroborate relationships between biomarkers, thus enabling accurate progression evaluation.

Much effort has been made to improve the detection sensitivity of SERS-based biosensors using molecules with a large Raman cross-section, including triarylmethane dyes (rhodamine 6 G) and methine dyes (cyanine 3). However, their signals overlap considerably with those signals from biological molecules in plasma^11^. Therefore, we believe that a carbon monoxide (CO) probe carrying signals in the spectral region of 2000 cm^−1^ could improve the detection sensitivity of MMPs because protein and DNA have no detectable Raman signals in the spectral region of 1780–2200 cm^−1 ^^12^. CO can form strong bonds with transition metals due to the strong *σ*− and *π*−acceptor features of CO. Thus, the use of a transition metal carbonyl could be the best option for biodetection^13–15^.

In this work, we report a unique clinical strategy that addresses the major limitations of effective biomarkers in monitoring NPC progression by Raman-based sensing. In addition, we developed a nanogel substrate (0.3-cm diameter) that enables the further enhanced and stabilized detection of Raman signals. A nanogel substrate has been carefully designed with the goal of trapping SERS nanoparticles that correspond to the proteolytic activity of MMPs, helping physicians access to low concentrations of MMPs of interest that could guide follow-up treatment.

## Results

### Tuning of totally symmetric frequency of metal carbonyls

**Fig. 1:**
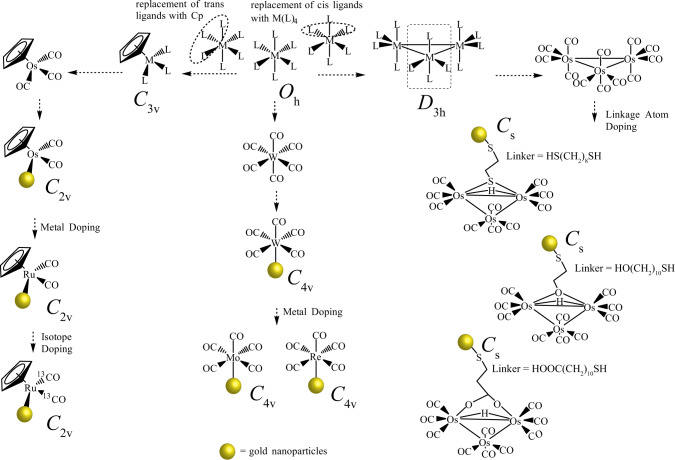
Geometrically encoded SERS nanobarcodes. Engineering of SERS nanoparticles via geometric control of metal carbonyl reporters.

**Fig. 2:**
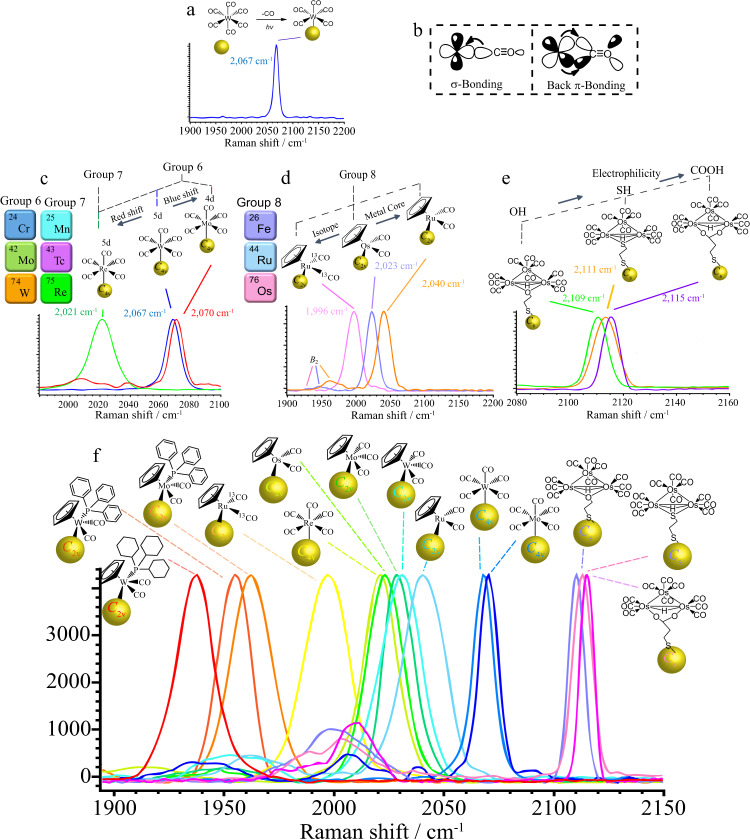
Multiplexing geometrically encoded SERS nanobarcodes. **a** Raman spectra of W(CO)_6_ on Au nanoparticles. **b** Schematic illustration of *σ* and back-*π* bonding interactions between the d orbitals of the metal and CO ligand. **c** Tuning of the totally symmetric mode (~2000 cm^−1^ region) of *C*_4v_ geometry SERS reporters by doping with various groups of transition metals, resulting in different levels of synergistic bonding effects. **d** Application of Cp as a *σ* ligand and isotope effect to tune the totally symmetric mode (~1900 cm^−1^ region) of *C*_2v_ geometry SERS reporters. **e** Linkage effect on fine-tuning of the totally symmetric mode (~2100 cm^−1^ region) of *C*_s_ geometry SERS reporters. **f** Multiplexed CO nanotags. SERS peaks of CO nanotags in the optical interference-free window. Source data are provided as a Source Data file.

The most prevalent coordination geometry in inorganic chemistry is octahedral (*O*_h_ symmetry), where six ligands are bonded to the transition metal atom in a symmetrical distribution (Fig. 1). Metal carbonyl complexes (M(CO)_6_) with *O*_h_ symmetry exhibit *A*_1g_ and *E*_g_ (g = gerade) Raman vibrations. Notably, due to the large extent of polarization, the *A*_1g_ band is always detectable and is one of the characteristic features of the Raman spectra of M(CO)_6_^16^. We first started with tungsten (W) metal carbonyl, W(CO)_6_, which is known to exhibit the *A*_1g_ band in Raman spectra^17,18^. It was conjugated to the gold (Au) surface via a photochemical reaction^19^. Upon irradiation, W(CO)_6_ is reported to dissociate one CO and subsequently adsorb on the Au surface as W(CO)_5_-Au (Fig. 2a)^19^. After the incorporation of W(CO)_6_ on Au nanoparticles, a strong Raman vibration band was detected and assigned as *A*_1_ (the g subscript was excluded due to the loss of centrosymmetry)^20^. The difference in the band assignment after the photochemical reaction was due to the lowering of symmetry from *O*_h_ to *C*_4v_ after binding on the Au surface^21^. More importantly, the totally symmetric mode displayed an intense Raman peak with a narrow line width (10 cm^−1^) (Supplementary Fig. 1). The narrow line width is important for improving spectral resolution for multiplexing detection and expanding the multiplexing library.

It is well known that the CO band position often correlates with the electron density donated to the CO π* orbitals, which is the *π*-donor ability of the metal centre (Fig. 2b). As greater electron charge from CO is directed into the empty π* orbital of carbonyl, the CO bond is weakened; this, in response, increases the bond order between metal and CO ligand^8,22^. Thus, the position of the metal in the periodic table plays a role. We replaced the metal centre with rhenium (Re) and molybdenum (Mo), which was expected to tune the CO stretching frequency (Fig. 2c). Compared with W, the totally symmetric mode of the A_1_ band of CO in the Raman spectra of Re(CO)_5_ and Mo(CO)_5_ moieties on Au nanoparticles shifted to 2021 cm^−1^ and 2070 cm^−1^, respectively. The degree of redshifting CO stretching frequency was greater for Re, which was attributed to the fact that Re possesses 5d orbitals that are larger than the 4d orbital of Mo, resulting in more effective overlap with the π* CO antibonding orbitals.

More complex metal carbonyl structures can often be obtained by using the pseudosymmetry of the M(CO)_n_ group, in which cyclopentadienyl (Cp) osmium (Os) tricarbonyl can be regarded as an M(CO)_3_ moiety with *C*_3v_ symmetry (Fig. 1). The conjugation of CpOs(CO)_3_ to Au nanoparticles was achieved by replacing a CO ligand with iodine and subsequently anchoring the CpOs(CO)_2_ moiety via abstraction of the iodide reaction^23–26^ (Supplementary Fig. 2). The CpOs(CO)_2_ bound on the nanoparticle surface was evidenced by energy dispersive X-Ray (EDX) analysis (Supplementary Fig. 3). The tuning of CO spectra by doping ruthenium into CpM(CO)_3_ was demonstrated recently^8,27^. The displacement of one CO ligand from CpM(CO)_3_ (M = Os or Ru) resulted in lowering the symmetry from *C*_3v_ to *C*_2v_^28^. Because the M(CO)_2_ moiety possesses *C*_2v_ local symmetry, two strong IR-active CO stretching modes (*A*_1_ and *B*_2_) are also expected (Supplementary Figs. 4 and 5). The Os and Ru nanotags showed a detectable weak SERS signal at ~1,900 cm^−1^ (*B*_1_) and a distinct signal at ~2,000 cm^−1^ (*A*_1_) (Fig. 2d). Compared to M(CO)_5_ on Au in Fig. 2c, the totally symmetric Raman stretching of the *A*_1_ band for the M(CO)_2_ moiety was redshifted by ~30 cm^−1^ due to the synergistic bonding effect whereby the charge density on the metal atom affects the *σ*-*π* and *σ*-π* processes. In addition, the Cp system is trans to the CO ligands and is a better σ donor and a weaker *π* acceptor than the CO ligand; the Cp group in fact stabilizes the M-C bonding, leading to a substantial lowering of the wavenumbers^27^.

We sought to further engineer the M(CO)_2_ moiety to expand the library of vibrational frequencies. We utilized carbon isotope doping (^13^CO), which could modulate the reduced mass to reduce the wavenumber of CO. Given that the force constants remain unchanged upon isotopic substitution, the frequencies corresponding to these two vibrations are related by the reduced masses of the ^12^CO and ^13^CO groups. Thus, for a ^12^CO oscillator with a stretching frequency of 2000 cm^−1^, the frequency of the equivalent ^13^CO oscillator will be ca. 45 cm^−1^ lower (Supplementary Fig. 6)^29^. Therefore, bands located approximately 45 cm^−1^ below those of the fully ^12^CO-substituted Ru (1996 cm^−1^) can be associated with the corresponding ^13^CO stretching vibration of a ^13^CO-substituted derivative (Fig. 2d). Thus, such ^13^CO doping allows us to tune the sharp frequency in a range of ~45 cm^−1^.

The replacement of two cis-ligands by other ligands in M(CO)_6_ will result in the *C*_2v_ point group of the metal carbonyl (M(CO)_4_(L)_2_) (Fig. 1)^30^. The combination of 3 units of *C*_2v_ can obtain a metal carbonyl with *D*_3h_ symmetry^31^. The best example is Os_3_(CO)_12_ (Os_3_), a very stable species. Os_3_ species under *D*_3h_ symmetry exhibit six Raman-active bands (2*A*_1_′ + *A*_2_′ + *A*_2_″ + 3*E*′ + *E*″)^32,33^. The Raman shift for the primary *A*_1_′ mode is 2127 cm^−1^ (Supplementary Fig. 7)^33^. However, a functional linker is required to immobilize Os_3_ species onto Au nanoparticles. We first exploited Os_3_ with a thiol ligand because thiol ligands are relatively soft and are good σ and π acceptors. Linker HS(CH_2_)_8_SH was selected as the linker due to its structural simplicity. Using an oxidative addition reaction accompanied by the dissociation of two CO ligands, thiolated triosmium carbonyl with thiol linkage groups was prepared (Os_3_-SH) (Supplementary Figs. 8, 9)^20,34^. However, removing the two CO lowers the symmetry from *D*_3h_ to *C*_s_^34^. According to the character table of *C*_s_, such species will possess an *A*_1_′ Raman-active band (Supplementary Fig. 10)^35^. The *A*_1_′ band in the high-frequency CO stretching vibration is also infrared active but is often of low intensity. Similar phenomena can also be observed in *D*_3h_ symmetry complexes^36^. The immobilization of Os_3_-SH on Au nanoparticles is confirmed by the detected fragmented species of Os_3_-SH (Supplementary Fig. 11). The totally symmetric mode of *C*_s_ was detected in the 2,100 cm^−1^ (for Os_3_-SH = 2111 cm^−1^) region, which is higher energy than the symmetric band of *O*_h_ complexes because the totally symmetric CO stretching vibration involves greater repulsion for CO in the Os_3_ structure.

Doping of oxo- (Os_3_-OH) and carboxylate (Os_3_-COOH)-functional groups into the linkage site results in totally symmetric stretching frequencies of CO shifting to 2109 cm^−1^ to 2115 cm^−1^, respectively (Fig. 2e and Supplementary Figs. 12 and 15). The thiol, hydroxyl and carboxylic groups occupy two axial positions on the triosmium plane^37,38^. Due to the electron density delocalized on the Os-Os edge-bridging bonding molecular orbitals (Supplementary Fig. 16)^39^, ligands with different *σ*-donating and *π*-accepting properties are expected to influence the electron density on the clusters. Thus, thiol, which is a soft base^40–42^ and a good *σ*-donor and back *π* acceptor, has a strong interaction between Os and CO. This observation is consistent with the reported crystal structures of Os_3_ species that the CO ligands trans to the thiol group are notably shorter than those in hydroxylated and carboxylated triosmium species^20,37,43,44^. Thus, a higher stretching frequency for the totally symmetric mode for carboxylated triosmium is always reported. Such single sharp peaks from Os_3_-SH, Os_3_-OH and Os_3_-COOH are in the region of 2,100 cm^−1^, which compensates for the SERS nanoparticle library and holds promise for multiplex detection.

Guided by the principles of organometallic chemistry, a library for CO frequency multiplexing was prepared. This library was combined with our previous findings to represent a large number of resolvable CO frequencies in the optical interference-free region (Fig. 2f and Supplementary Fig. 17)^8,27^. Notably, the signal-to-noise ratio is high overall. The TEM results show that these nanotags are uniformly dispersed without aggregation even after 30 days (Supplementary Fig. 18).

### Nanogel leaning nanopillars substrate preparation

**Fig. 3:**
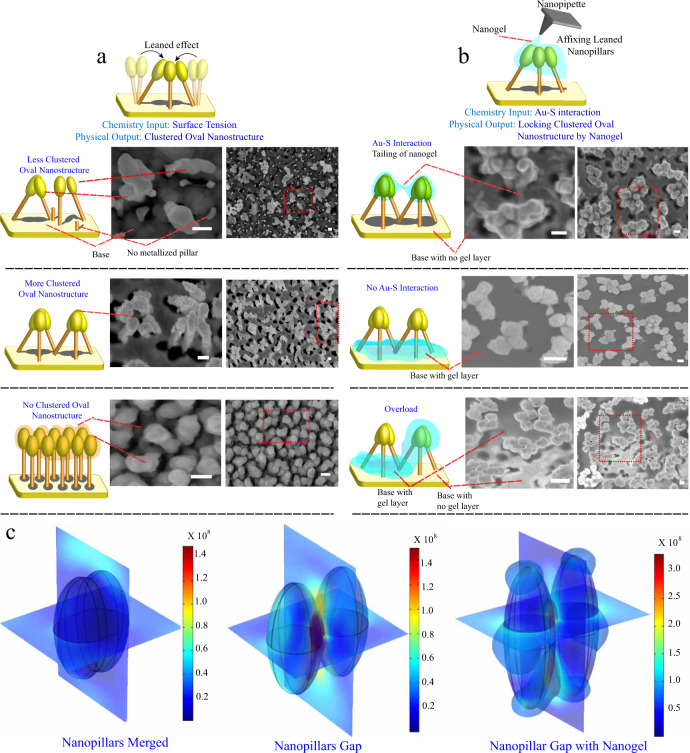
Deposition nanogel on fabricated nanopillars. **a** The effect of the level of Au deposition on the formation of leaning nanopillars. A representative image of three individual experiments is shown (scale bar: 100 nm). **b** The importance of Au-thiol interactions for affixing leaning nanopillars on nanogels. A representative image of three individual experiments is shown (scale bar: 100 nm). **c** The importance of the gap between nanopillars and the deposition of nanogel shows no significant disruption to the electromagnetic wave of nanopillars.

Nanoparticle aggregation on a SERS-active substrate could produce strong SERS enhancement from the inter-nanostructure coupling under visible light radiation. For this purpose, an in-house fabricated SERS-active substrate (0.3 cm × 0.3 cm size) containing a nanopillar array structure is used. We washed the nanopillar substrate with water, which can induce surface tension among nanopillars to form nanosized nanopillar clusters by leaning towards one another (Fig. 3a). To obtain maximum hot spot generation, the metal deposition process is critical. A substrate lacking metallization of Au resulted in the formation of significantly fewer leaning nanopillars (Fig. 3a). On the other hand, with overmetallization of Au, no leaning process can occur due to the merging of oval nanostructures (Fig. 3a). We found that a deposition velocity of 3 and 10 Å/s for chromium and silver (at 2 × 10^−6^ mbar pressure) will give a substrate with a high density of leaning nanopillars (Fig. 3a). These aggregated nanopillars are the result of bending of silicon pillars (Supplementary Fig. 19). As demonstrated by the electromagnetic (EM) field simulation, there was a strong EM field for leaned nanopillars (Fig. 3c) and a reduction in the intensity of the EM field once the nanopillars merged (Fig. 3c).

We functionalized nanopillars with nanogel to increase the steadiness of the hotspot, as shown in Fig. 3c, where the simulated EM field remained strong after deposition of the nanogel on the surface. The nanogel was derived from poly(BAC-AMPD)s and prepared by the Michael addition polymerization of 4-(aminomethyl)piperidine (AMPD) and N,N-cystaminebis(acrylamide) (BAC) (Supplementary Fig. 20). Fountain pen nanolithography (FPN) was utilized to deposit nanogels to nanopillars (Supplementary Figs. 21 and 22). The drawing speed is microns per millisecond. In Fig. 3b, a slow speed (0.02 μm/ms) and 10 nm nanogel produced a nanogel fixed leaning nanopillar structure. Nanogel tailing can be observed between interleaning nanopillar clusters that have a width of ~20 nm. The same probe with nanogel without a free sulfhydryl group (no lipoic acid) produces nanogel deposited on the base of the substrate due to lack of S-Au interaction (Fig. 3b). As shown in Fig. 3b, the concentration (100 nM) was ten times the amount of dispersion. The overflow of the nanogel is shown, with a bundle of nanogel tailings on the leaning nanopillar and a significant amount of nanogel deposited on the bottom of the substrate. The factors that control the drawing of nanogels on leaned nanopillars depend on optimization of the concentration and the settling time to form binding of the sulfhydryl group. The nanogel was allowed to settle for 24 h in a dry box.

### Boolean logic SINGLE detection by cleave-bind-and-trap sensing mechanism

**Fig. 4:**
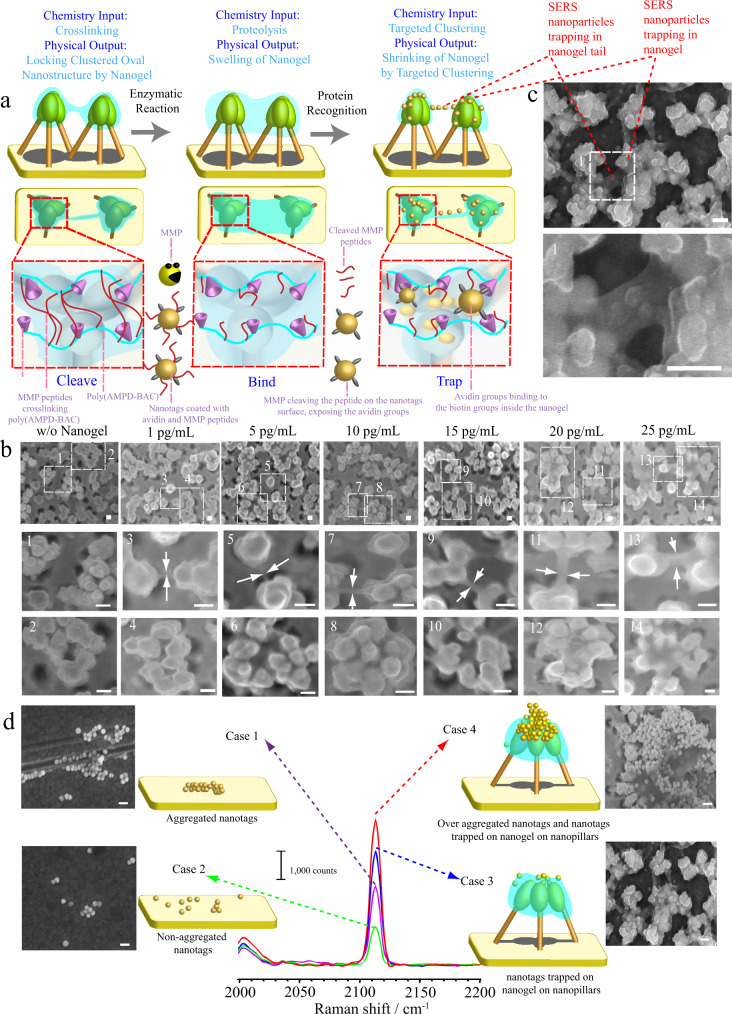
Study of cleave-bind-and-trap detection mechanism. **a** Schematic illustration of the cleavage-binding-and-trap detection mechanism involving MMP peptide cleavage, biotin-avidin recognition and SERS nanoparticles trapped in nanogel. **b** A series of SEM experiments demonstrating nanogel swelling associated with the concentration of MMP-2. **c** Trapping process of nanotags in nanogel as evidenced by SEM images. **d** CO intensity enhanced by nanotags, aggregated nanotags and clustered nanotags on nanopillars. A representative image of three individual experiments is shown (scale bar: 100 nm).

For cleavage-binding-and-trap mechanism detection (Fig. 4a), Os_3_-SH SERS nanoparticles on the nanogel leaning-nanopillar structures actuate in the presence of MMP-2. MMP-2 expression contributes to tumor aggressiveness and poor prognosis and induces tumor occurrence^45–47^. For the cleavage-binding-and-trap mechanism, the MMP-2 peptide substrate encoded with Os_3_-SH SERS nanotags (Supplementary Fig. 23) and coated with avidin served as a binder, and biotin on the nanogel-leaning nanopillar crosslinked with the respective MMP peptide served as a receptor (Fig. 4a). This design can ensure that in the absence of MMP-2, avidin-functionalized nanotags are unable to bind to biotin on the nanogel.

The nanogel-leaning nanopillar acts as a trapping platform, which is expected to allow SERS nanotags to be trapped over the leaning nanopillar on the nanogel layer. The interaction of SERS nanotags with the biotin embedded on the nanogel after removing the MMP peptide is a plausible mechanism for nanoparticles trapped and clustered on the nanogel layer, which involves dimensional changes in the nanogel upon cleavage of the MMP-2 peptide and binding with biotin-derivative SERS nanoparticles in the nanogel. This was further evidenced by nanogel swelling after incubation with the MMP-2 enzyme (Fig. 4b). This is because we did not observe nanogel swelling on the nanopillar in the absence of the MMP-2 enzyme. In contrast, the level of swelling of the nanogel increased as the concentration of MMP-2 increased. Upon adding avidin-coated SERS nanotags, we observed clustered SERS nanotags trapped within the nanogel, resulting in shrinkage of the nanogel because the SERS nanotags crosslinked via the interaction of avidin and biotin when SERS nanotags were trapped at the nanopillar tips (Fig. 4c).

Figure 4d depicts the Os_3_-SH SERS nanotags aggregated around the nanopillar array structures to achieve greater enhancement of signals. Compared with aggregated (Case 1) and nonaggregated SERS nanotags (Case 2) on the Au surface, the aggregation of SERS nanotags on the nanogel with leaning nanopillars (Case 3) led to a ~2-fold enhancement of signals. If the degree of clustering greatly increased (Case 4; a dense aggregation of nanotags on the nanogel over the leaning nanopillars), the CO signals were further enhanced. However, signal fluctuation was observed for the signal generated by such a thick layer of nanotags because the nanotags were not trapped and stabilized within the nanogel. For Case 3, the clustered nanotags could still be spotted 6 months later (Supplementary Fig. 24), and no significant signal loss was observed, which demonstrated that the nanogel can not only stabilize the leaning nanopillar but can also preserve the SERS nanotags for a long period of time, which is useful for clinical record keeping.

### Boolean logic AND detection

It was also reported that MMP-1 is the most important factor in cancer development. Many studies have demonstrated that MMP-1 increases gradually from clinical stage I to stage IV^48^. Additionally, upregulation of MMP-1 has been demonstrated to be correlated with lymph node metastasis in some cancers, such as nasopharyngeal carcinoma (NPC). Thus, the detection of biomarkers of MMP-1 and MMP-2 that can cover the early stage and late stage of monitoring would be desirable.

**Fig. 5:**
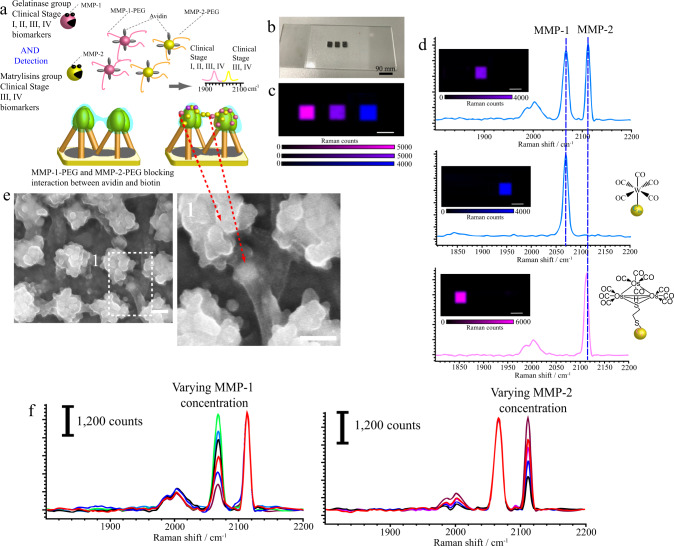
Boolean logic AND detection. **a** Schematic representative of Boolean logic AND detection of MMP-2 and MMP-1. **b** Images of the nanogel substrate on a quartz slide. **c** SERS nanotags on a nanogel substrate spatially separated onto a piece of quartz slide. **d** Raman spectroscopy to unmix two colocalized symmetric bands of SERS nanotags on a nanogel substrate. The resulting images show the two SERS nanotags correctly separated into their corresponding spectral channels with minimal cross-talk between the spectra. **e** Trapping process of nanotags in nanogel as evidenced by SEM images. A representative image of three individual experiments is shown (scale bar: 100 nm). **f** Detection of various concentrations of MMP-1 (0.5–10 ng/mL) in the presence of 5 ng/mL MMP-2 and detection of MMP-2 (0.5–10 ng/mL) in the presence of 10 ng/mL MMP-1. Source data are provided as a Source Data file.

In the logical AND system, we will demonstrate MMP-2 (gelatinase group) peptide-PEG encoded with Os_3_-SH SERS nanotags. This will pair with W(CO)_5_ SERS nanotags MMP-1 peptide-PEG (matrylisin group). This design can ensure that in the absence of the respective MMP, avidin-functionalized nanotags are unable to bind to biotin on the nanogel (Fig. 5a). The molecular weight of PEG is 5000 Da, which is able to separate nanotags from aggregation^49,50^. The number of peptide-PEG per nanotag was estimated to be 1100 ± 320 (Supplementary Fig. 25), which is lower than the usual value^49^, because the surface of the Au nanoparticles was already shielded with metal carbonyl and avidin.

In the absence of specific proteases, the attached peptide-PEG on the nanotags could prevent the avidin on the nanotags from interacting with the biotin in the nanogel. However, only in the presence of the respective pair of proteases were the attached PEG polymers completely removed, permitting avidin (SERS nanotag)-biotin (substrate) interactions. Specifically, two CO signals could be observed on the nanogel, as also demonstrated in the SERS mapping (Fig. 5d).

We mapped the substrate on a quartz slide using 30-mm x/y translations at times of 1 s per spectral acquisition (Fig. 5b). The map was analysed with Wire Renishaw software. The mapping function could demonstrate the ability of Raman spectroscopy to unmix two colocalized symmetric bands of SERS nanoparticles. The resulting images in Fig. 5c, d show that the two SERS nanoparticles precisely separated into two spectral channels. Both logical tests can therefore serve as a quick test to monitor the progress of patients by detecting two classes of prognostic biomarkers. Nanotags trapped within the nanogel are also observable from the SEM images (Fig. 5e). This evidence indicates that the nanotags are also trapped within the tail of the nanogel. Intensity changes were detected while varying either the MMP-1 or MMP-2 concentration (Fig. 5f), demonstrating that the intensity of the nanotags corresponded well with the concentration of the respective MMP.

### Boolean logic OR detection

It is well known that overexpression of MMP-9 in NPC could facilitate local invasion and tumor growth, indicating that MMP-9 plays a role in the progression of NPC tumor invasion and metastasis. Moreover, a study revealed that a high level of MMP-9 was also related with lymph node metastasis^13^. A study also found MMP-3 to be upregulated in NPC patient serum compared with control serum, indicating that MMP-3 could be a potential prognostic biomarker for NPC. Elevated MMP-7 expression was associated with lymph node metastasis but had no significant correlation with tumor stage or differentiation. Several studies have demonstrated that a high level of MMP-7 is connected to tumorigenesis and lymph node metastasis of tongue cancer. Therefore, a multibiomarker approach may be reasonable for the detection of biomarkers of NPC.

**Fig. 6:**
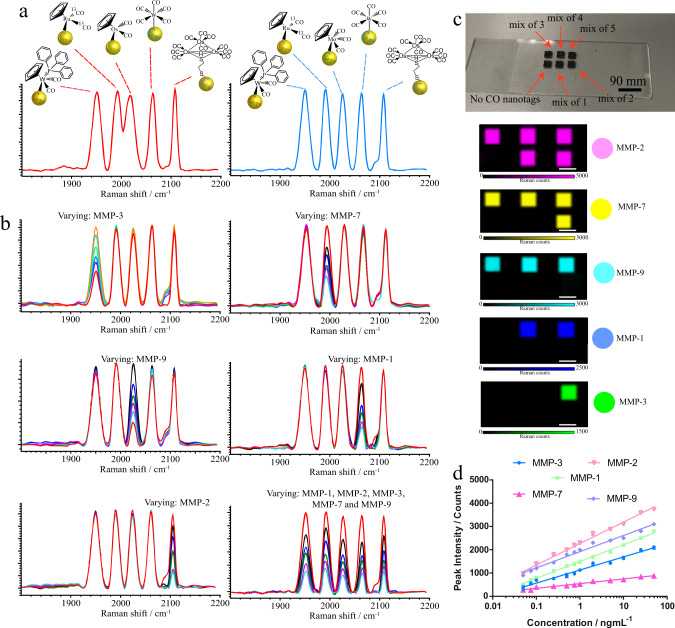
Boolean logic OR detection. **a** Spectra of metal carbonyls selected for evaluating the feasibility for multiplex sensing applications. **b** Selectivity of the SERS sensor towards various individual MMPs. **c** Demonstration of multiple colocalized SERS detection including mixtures of 1, 2, 3, 4 and 5 SERS nanotags within the same substrate. **d** Plot represents the intensity of the CO stretching frequency for multiple nanotags. Source data are provided as a Source Data file.

In this system, for a logical OR, clustering of SERS nanotags on the nanopillar structures actuates in the presence of MMP-1, MMP-2, MMP-3, MMP-7, or MMP-9, which is accomplished by MMP-1, MMP-2, MMP-3, MMP-7, and MMP-9 peptide substrates consisting of cleavage motifs in series. We selected SERS nanotags with moieties of W-PPh_3_(CO), Ru-^13^CO, Os-CO, Mo-CO, W-CO and Os_3_-SH for the evaluation of the multiplexing application. We found that the presence of Os-CO could increase the spectral cross-talk for multiplexing detection (Fig. 6a). The replacement of Os-CO with Mo-CO resulted in better resolvable optical spectra (Fig. 6a). We therefore selected W-PPh_3_(CO), Ru-^13^CO, Mo-CO, W-CO and Os_3_-SH for multiplex sensing and coated them with MMP-PEG. As peptides on MMP-PEG have thiol-containing amino acid cysteines, the coating process can be achieved via thiol-gold interactions. The number of metal carbonyls per Au NP was determined to be ~1,000 (Au NP (W-PPh_3_(CO)) = 636 ± 150, Au NP (Ru-^13^CO) = 765 ± 212, Au NP (Os-CO) = 920 ± 211, Au NP (W-CO) = 614 ± 111 and Au NP (Os_3_-SH) = 1632 ± 142). PEG is reported to endow gold nanoparticles with better dispersion stability. The dispersion stability was examined by dynamic light scattering (DLS). The conjugation of MMP-PEG was confirmed by an increase in hydrodynamic values compared to bare Au NPs for nanotags. The increase in hydrodynamics could be attributed to the adsorption of the metal carbonyls and the MMP-PEG peptide layer (Supplementary Fig. 23).This shows the good dispersion stability of Au NP nanotags even after 1 month of storage at 4 °C.

Nanotags were incubated with MMP enzymes and incubated on a nanopillar substrate, resulting in multipeak CO signals. Similarly, changes in the CO signal were easily observed by varying the respective MMP concentration (Fig. 6b), indicating that the multiplexed SERS sensor can selectively detect MMP without interference from other nontarget MMPs. The selectivity of the SERS sensor to each MMP was calculated based on the fluctuation of the SERS spectrum in the presence of nontarget MMPs (MMP-1 = 92%; MMP-2 = 96%; MMP-3 = 91%; MMP-7 = 93% and MMP-9 = 91%). We then successfully unmixed colocalized mixture of these five nanotags (Fig. 6c and Supplementary Fig. 26). By varying the five individual MMPs, we were able to obtain concentration-dependent spectra and plots (Fig. 6b and Fig. 6d).

Linear dependencies of the CO signal on the target MMP concentrations were observed (MMP-1: *R*^2^ = 0.99; MMP-2: *R*^2^ = 0.99; MMP-3: *R*^2^ = 0.99; MMP-7: *R*^2^ = 0.99 and MMP-9: *R*^2^ = 0.98). The limit of detection of MMPs using this method was determined to be 0.07 ng/mL for MMP-1, 0.083 ng/mL for MMP-2, 0.1 ng/mL for MMP-3, 0.14 ng/mL for MMP-7 and 0.11 ng/mL for MMP-9. With reference to the plot, it was observed that the detection range of our platform was from 0.1 ng/mL to 50 ng/mL (Supplementary Fig. 27). In addition, the SERS nanotag sensors exhibited a low chip-to-chip variation (an average CV of 6.1%) and batch-to-batch nanotags (an average CV of 9.0%) (Supplementary Table 1). The selectivity of detection was evaluated in the presence of interfering substances, and the recovery range was found to be > 92% (MMP-1: 93% MMP-2: 95%; MMP-3: 93%; MMP-7: 95% and MMP-9: 92%). Thus, nanotags and nanopillars had a distinct selectivity for multiplexed detection of MMPs over other species that were tested.

### Boolean logic detection for nasopharyngeal carcinoma clinical species

It is well known that the current testing kit that is used in clinical practice is one MMP one test kit basic. However, such testing is labour intensive and expensive for multiplexing detection. Moreover, such tests are relatively complex and require a large sample volume. In contrast, readout and quantitative measurements of the MMP concentration by SERS using a nanogel substrate require only a 0.3 × 0.3 cm^2^ substrate with multiplexing nanotags, which renders this approach simple, low-cost, and practical. Patient response to MMPs concentration was monitored. The nanogel substrate could reduce the volume of blood sample significantly to approximately 5–10 μL, which offers a fast sample collection time (approximately 3 s) using a needle poke instead of a syringe. The response times for multiplexing detection are impressive compared to those of clinical kits (ELISA test: volume of blood withdrawn is ~ 5 mL, the blood collection time is ~ 10 min and minimum required volume for 96-well ELISA microplates is 100 µL for each MMP test). While an ELISA can be run in the same amount of time that the SERS assay took and the 96 wells can be used to screen for numerous biomarkers, the SERS assay can be potentially miniaturized and uses very little sample volume. Another advantage is the miniaturization of these sensors for more than 5 MMP biomarkers. Moreover, we believe that this technology could potentially be developed for larger arrays.

**Fig. 7:**
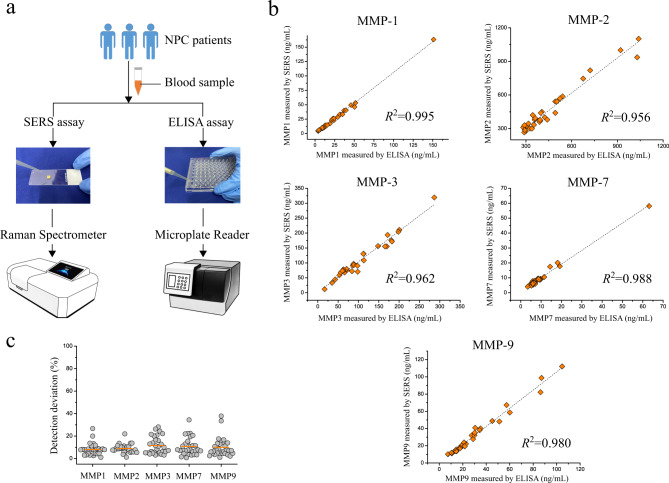
Performance of SERS sensor. **a** Schematic of MMP detection using SERS and ELISAs. **b** Concordance between concentrations of MMP measured by SERS and ELISAs for MMP-1, MMP-2, MMP-3, MMP-7 and MMP-9. **c** Scatter plots of the detection deviation for SERS and ELISA tests. The orange line represents the mean value.

To demonstrate the potential application of this MMP sensing method in clinical scenarios, the proposed sensing platform based on SERS was applied to quantify five MMPs (MMP-1, MMP-2, MMP-3, MMP-7 and MMP-9) in human peripheral blood samples from 30 NPC patients. Meanwhile, the same blood samples were measured by commercial ELISA kits to verify the accuracy of the SERS assay for MMP detection. Figure 7a demonstrates the schematic of MMP detection in clinical human blood samples using SERS and ELISAs. The results can be seen and compared in Supplementary Table 2 and Supplementary Table 3. By analysing the concordance between concentrations of MMPs measured by the two different assays (Fig. 7b), it was found that the level of MMPs determined by the SERS method was strongly associated with that assessed by ELISA, with the *R*^2^ of 0.995 for MMP-1, 0.956 for MMP-2, 0.962 for MMP-3, 0.988 for MMP-7 and 0.980 for MMP-9, respectively. Furthermore, the detection deviation is calculated, with mean values of only 8.05%, 8.92%, 11.56%, 10.74% and 9.91% for MMP-1, MMP-2, MMP-3, MMP-7 and MMP-9 detection, respectively. These results demonstrate the high feasibility of this SERS-based sensing platform for MMP detection in clinical human blood samples. In comparison to the conventional ELISA, this SERS is capable of achieving multiplexed detection and comparable detection accuracy for MMPs while associated with less blood sample volume and convenient procedure, enabling it to be an alternative method for monitoring various MMPs in clinical practice.

**Fig. 8:**
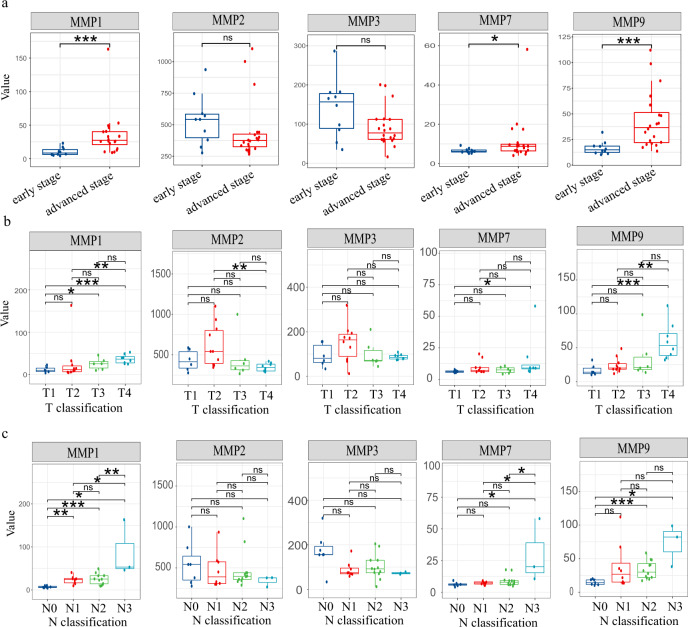
Correlation between the clinical pathological parameters and MMPs level detected by the SERS sensor. Box plot presenting the differential MMP levels in various clinicopathologic parameters, including **a** stage (early stage patients, *n* = 10; advanced stage patients, *n* = 20), **b** T classification (T1 patients, *n* = 6; T2 patients, *n* = 10; T3 patients, *n* = 6; T4 patients, *n* = 8), and **c** N classification (N0 patients, *n* = 7; N1 patients, n = 8; N2 patients, *n* = 12; N3 patients, *n* = 3). T and N represent the tumor and nodes information, respectively. ns: no significance. *P < 0.05. **P < 0.01. ***P < 0.001. The exact *P* values for each combination are summarized in Supplementary Table 4. The line within each notch box represents the median, and the lower and upper boundaries of the box indicate the first and third quartiles respectively. The whiskers represent the 1.5x interquartile range. Two-sided Student’s *t*-test was used for statistical analysis, and no adjustments were made multiple comparisons.

Cancer progression and metastasis are associated with extracellular matrix responses. Therefore, matrix metalloproteinases, in this regard, have received tremendous attention during cancer development^51,52^. MMPs play important roles in degrading multiple components of the extracellular matrix^53^. This is critical for remodelling the ECM, thereby contributing to tumor progression, invasion and metastasis. MMP family members, as secreted proteins, have distinct serum levels and are potential promising biomarker markers for disease monitoring^54^. Therefore, we analysed the correlation between the MMP level and clinicopathological parameters, including clinical stage (Fig. 8a), T classification (Fig. 8b), and N classification (Fig. 8c), in NPC patients.

A previous study demonstrated that MMP-1 overexpression was detected in NPC patients, and MMP-1 expression was significantly associated with advanced T stage and poor survival of NPC^55,56^. Consistent with these reports, our results revealed that MMP-1 was remarkably correlated with clinicopathological parameters (e.g., advanced T classification, N classification, and clinical stage) of NPC patients. MMP-2 and MMP-3 have the ability to degrade collagen II, IV, IX, X, XI, and gelatine, which is known to be critical for tumor invasion and metastasis^47,57^. However, in the current study, we failed to observe a significant relationship between MMP-2 and MMP-3 levels and advanced T classification, N classification, and clinical stage. In contrast to MMP-2 and MMP-3, high MMP-7 levels were frequently detected in advanced clinical stage NPC patients in this study, suggesting a possible role for MMP-7 in the determination of advanced cancer in NPC. Maurel et al. also found that MMP-7 levels in human blood samples were significantly elevated in patients with advanced colorectal cancer, owing to the important role of MMP-7 in tumor growth, invasion and dissemination^58^. Similar to MMP-7, our results showed that a high MMP-9 level was significantly associated with advanced T classification, N classification, and clinical stage, which is consistent with the study conducted by Liu et al.^59^.

**Fig. 9:**
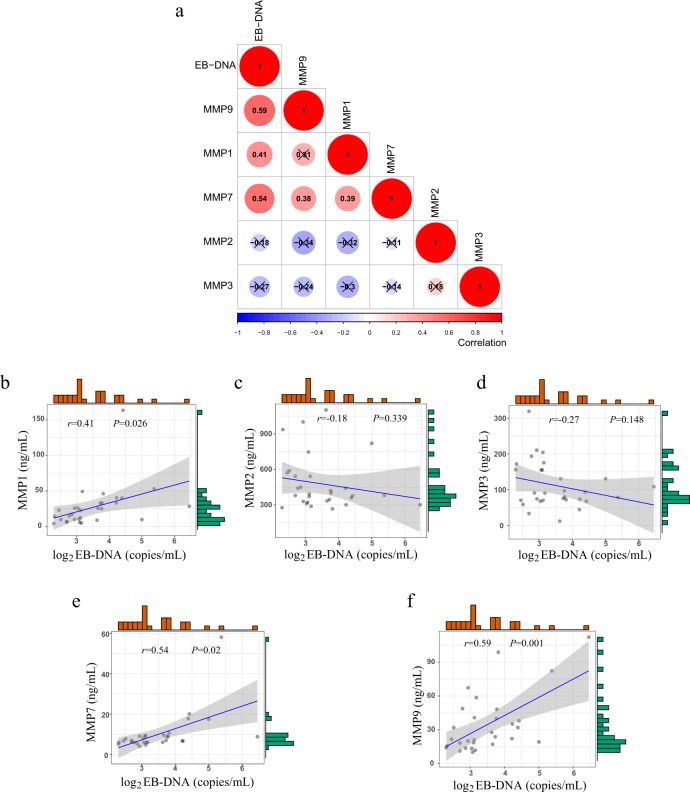
Clinical relevance of MMPs and EBV-DNA levels for NPC patients. **a** Heatmap visualizing the correlation of EBV-DNA level with each MMP. Scatter plots presenting the correlation of EBV-DNA level with **b** MMP-1, **c** MMP-2, **d** MMP-3, **e** MMP-7, and **f** MMP-9. The histograms represent the frequency distribution of MMP and EBV level. The correction coefficient (*r*) was obtained by performing the Pearson’s correction analysis, and no adjustments were made multiple comparisons.

Epstein-Barr virus (EBV) is a promising biomarker in NPC. Quantitative analysis of plasma EBV-DNA is used for population screening, prognostication, and disease surveillance^60,61^. Here, we further analysed the correlation of EBV-DNA levels with MMPs. Figure 9a presents the correlation among EBV-DNA level, MMP-1, MMP-2, MMP-3, MMP-7, and MMP-9. As shown in Fig. 9b, e and f, MMP-1, MMP-7, and MMP-9 were positively associated with EBV-DNA levels. Although MMP-2 and MMP-3 levels showed a negative correlation tendency with EBV-DNA levels, the *P*-value was not significant (Fig. 9c and d). EBV infection and genetic susceptibility are two main risk factors for NPC development^61^. Previous studies^62,63^ have supported that EBV-associated proteins will upregulate the expression level of MMPs, thereby conferring the invasive characteristics of the cells. In line with this evidence, our study revealed that plasma EBV-DNA levels were positively correlated with MMP-1, MMP-7, and MMP-9 levels. This indicated that EBV may promote tumor progression via upregulation of MMPs.

## Discussion

We created a palette of metal carbonyl-conjugated nanotags with each displaying a single and sharp band in the silent spectral window. With proper geometric control, we deliver 14 resolvable spectra, with the prospects for further development. The SERS sensor exhibited high reliability (an average CV of 6.1% for chip-to-chip variation and an average CV of 9.0% for batch-to-batch nanotags). It also exhibited the highest sensitivity (an average LOD of 0.1 ng/mL) when compared with reported MMP sensors (Supplementary Table 5). The superior sensitivity and low variation (CV) enabled the high precision detection of five MMPs. The LODs of the colorimetric assay and potentiometric detection were 10^3^-fold higher than those of our SERS sensors. Using the SERS sensor concept, simultaneous detection of five MMPs on a chip is highly achievable and readily integrated into a portable device.

According to the results, MMP-1 (*P*-value = 0.026), MMP-7 (*P*-value = 0.02), and MMP-9 (*P*-value = 0.001) are accurate biomarkers in monitoring NPC progression and are positively correlated with the level of EBV-DNA in plasma. Elevated MMP-7 levels were frequently detected in advanced clinical stage NPC patients in this study. Moreover, high MMP-9 levels are significantly associated with advanced T classification, N classification, and clinical stage. Such multiplex detection of MMPs is highly desirable because it can be more comprehensive to predict the progression of cancer.

With the support of the nanogel, the SERS nanotag remained clustered on the nanopillars over time for MMP detection, and no significant signal loss was observed over 6 months (Supplementary Fig. 24), which demonstrated that the nanogel not only stabilizes the leaning nanopillar but also preserves the SERS nanoparticle signal for a long period of time, which is useful for maintaining clinical records.

In summary, complexes of metals with CO ligands have promising applications in biotechnology. The outcomes from our study further demonstrate the feasibility of the approach for clinical use.

## Methods

### General procedure

All chemical reactions were carried out under a nitrogen atmosphere with Schlenk line equipment. IR spectra were collected on a Thermo Scientific Nicolet iS5N FT-NIR spectrometer. The Raman measurements were carried out using a Renishaw inVia Raman (UK) microscope equipped with a Peltier cooled CCD detector at an excitation laser wavelength of 785 nm (exposure time = 10 s). Subsequently, WiRE 4.3 software was used to perform polynomial multipoint fitting analysis, curve fitting analysis, and baseline correction for spectral data. The intensity of Raman peaks was also obtained from peak height analysis using WiRE 4.3 software. SERS 2D mapping was performed with a MS 20 ENCODED stage in Renishaw inVia Raman microscope. For each 2D mapping, ~ 3600 SERS spectra were produced (scan area = 60 μm × 60 μm; step size = 1 μm). TEM and SEM images were obtained using Hitachi H-7100 and KEOL-JSM-7600F, respectively. TEM samples were obtained by deposition of a drop of nanotags onto 400 mesh carbon copper grids.

### Preparation of W(CO)_5_ on gold nanoparticles

Ten milliliters of ethanolic solution containing 2.6 × 10^11^ particles of 60 nm gold colloids (BBInternational UK) were mixed with 1 mM W(CO)_6_. The solution in a 4-cm quartz cell was irradiated with a photolysis light source (Philips HPK 125-W medium-pressure mercury arc). After irradiation for 12 h, the solution was centrifuged at 177 × g for 1 min. The formed pellet was collected and washed with 1 mL of ethanol three times.

### Preparation of Re(CO)_5_ and Mn(CO)_5_ on gold nanoparticles

Re(CO)_5_Cl (Sigma Aldrich) or Mn(CO)_5_Cl was obtained from Sigma Aldrich. Freshly prepared ethanolic solution of Re(CO)_5_Cl or Mn(CO)_5_Cl (100 μM, 1 μL) was mixed with 10 mL of gold colloidal solution containing 2.6 × 10^11^ particles (BBInternational UK) in ethanol. After 24 h of incubation at 28 °C, the nanoparticles was collected by centrifugation at 177 × g for 1 min, and washed with 1 mL ethanol for three times.

### Preparation of CpM(CO)_2_-Au nanotags (M = Os and Ru)

In a typical reaction, 60 nm gold nanoparticles (3 mL, 2.6 × 10^10^ particles/mL) were centrifuged and redispersed in ethanol (990 μL). Freshly prepared solution of CpOs(CO)_2_-I or CpRu(CO)_2_-I (10 μL, 10 mM in ethanol) was mixed with 990 μL of gold nanoparticles and incubated at room temperature for 2 h. Excess complexes were removed by centrifugation at 177 × g for 2 min. The conjugated pellets were resuspended in deionized water (100 μL). An aliquot (20 μL) of the solution was dropped onto a glass slide and scanned under a Raman microscope (excitation wavelength = 785 nm, exposure time = 10 s and scan accumulation = 3). For the preparation of isotope labelling of CpRu(CO)_2_I,^64^ CpRu(CO)_2_Cl (0.150 g, 0.54 mmol) was first dissolved in 3 mL of n-decane. The resultant solution was stirred for 2 h at 140 °C while pure CO (1 atm) gas was supplied. The CpRu(^13^CO)_2_I was purified by passing through a silica gel column with hexane as eluent.

Yield: 0.103 g (74.2%).

^1^H NMR (CDCl_3_): *δ* 5.43 (s, 5H, (C_6_H_5_)). ^13^C NMR (CDCl_3_): *δ* 195.83, 87.31.

### Preparation of Os_3_(CO)_10_(*μ*-S(CH_2_)_8_S-Au nanotag

Os_3_(CO)_10_(NCCH_3_)_2_ (200 mg, 0.21 mmol) was mixed with 1,8-octandithiol (74 mg, 0.42 mmol) in methylene chloride (30 ml). The mixture was stirred for 12 h at room temperature. The solution was then concentrated using vacuum rotary evaporator. A yellow oil was obtained after thin-layer chromatography (hexane/ethyl acetate 1:1).

*ν*CO (DCM): 2108w, 2066 s, 2057w, 2020 s, 1996m cm^−1^.

^1^H NMR (CDCl_3_): *δ* 2.50 (q, CH_2_SH), 2.35 (t, SCH_2_), 1.63 (m, SCH_2_CH_2_, CH_2_CH_2_SH, SH), 1.30–1.47 (m, SCH_2_CH_2_(CH_2_)_4_CH_2_CH_2_SH), −17.40 (s, OsHOs)

After that, 10 μL of Os_3_(CO)_10_(*μ*-S(CH_2_)_8_SH (1 mM in ethanol) was mixed with 1 mL of gold nanoparticles (2.6 × 10^10^ particles/mL). The mixture was incubated for overnight. After 1 day of incubation, excess Os_3_(CO)_10_(*μ*-S(CH_2_)_8_SH) was removed by centrifugation at 177 × g for 1 min. The pellet washed three times with 1 ml ethanol.

### Preparation of Os_3_(CO)_10_(*μ*,*η*^2^OOC(CH_2_)_10_S-Au nanotag and Os_3_(CO)_10_(*μ*-OC(CH_2_)_10_S-Au nanotag

10 μL of 11-Mercaptoundecanoic acid (0.1 mM in ethanol) or 11-Mercapto-1-undecanol (0.1 mM in ethanol) was mixed with 1 mL of gold nanoparticles (2.6 × 10^10^ particles/mL) overnight. After 1 day of incubation, excess 11-mercaptoundecanoic acid was removed by centrifugation at 177 × g for 1 min. The pellet washed three times with 1 ml ethanol. The 11-mercaptoundecanoic acid-coated gold nanoparticles were resuspended in 100 μL of acetonitrile. Os_3_(CO)_10_(NCCH_3_)_2_ (10 mg) was added to 11-mercaptoundecanoic acid-coated gold nanoparticles solution. The mixture was incubated for overnight at room temperature. After 1 day of incubation, excess Os_3_(CO)_10_(NCCH_3_)_2_ was removed by centrifugation at 177 × g for 1 min. The Pellet was resuspended in deionized water (100 μL).

### Determination of metal carbonyl on gold nanoparticles

To determine the concentration of metal carbonyl on gold nanoparticles (Supplementary Fig. 28), a gold nanoparticles pellet (4.3 × 10^−5^ μM) was suspended to DI water (100 μL). On the other hand, a stock solution of metal carbonyl (1 mM in in ethanol) was prepared. 10 μL of metal carbonyl from the stock solution was added into gold nanoparticles solution. The resulting solution was vortexed for 1 min. The mixture was incubated for 2 h. A pellet was separated from the supernatant and washed three times with 1 mL of DI water. The concentration of metal carbonyl was estimated by measuring the IR absorbance at ~2,000 cm^−1^.

### Fabrication of nanopillar array chip

A *p*-type single side polished silicon <100> wafers (4 inches) was first washed with BOE (NH_4_F/HF/H_2_O) and moisture was then removed by baking the wafer in oven (5 min; at 120 °C). A reactive ion etching system (RIE-10N, SAMCO) was used for the etching process. The wafer was cleaned with O_2_ plasma (PDC-001 cleaner from Harrick Plasma, 2.0 × 10^−1^ Torr vacuum). Argon plasma was applied (1 min) to eliminate the etchant gas residuals and the side products. Chromium was deposited by E-beam evaporation (FULINTEC E-GUN). To obtain 0.17 nm thickness of chromium, deposition velocity was set to be 0.1 Å/s. After that, a layer of gold (thickness = 180 nm) was coated on the wafer surface (evaporation rate = 1.0 Å/s). The wafer was ready for dicing into 3 mm × 3 mm chips (DS-150 II from Everprecision Tech).

### Synthesis of poly(BAC-AMPD)s

BAC (N,N‵-bis(acryloyl)cystamine from Polysciences, 6.96 g (26.7 mmol) was mixed with AMPD (4-aminomethyl piperidine from Alfa Aesar, 3.08 g, 26.7 mmol) in anhydrous methanol (40 mL). The mixture was stirred under argon atmosphere at room temperature for 30 days. To complete the reaction, 0.03 g (0.27 mmol) of AMPD was added into the resultant solution, and then stirred for 24 h. Ultrafiltration was performed using a dialysis membrane (molecular weigth cut-off = 2000, 1 liter of methanol).

Yield = 6.60 g (70%).

^1^H NMR (CH_3_OD): *δ* 1.24 (m, 2H, CH_2_), 1.52 (bs, 1H, CH), 1.75 (d, 2H, CH_2_), 2.03 (t, 4H, CH_2_), 2.38 (t, 4H, CH_2_), 2.49 (d, 2H, CH_2_), 2.65 (t, 2H, CH_2_), 2.83 (t, 4H, CH_2_), 2.95 (d, 2H, CH_2_), 3.49 (t, 4H, CH_2_).

### Preparation of poly(BAC-AMPD)-*g*-PEG

Poly(BAC-AMPD) (6.78 g (18 mmol) was mixed with monomethyl PEG 4-nitrophenyl carbonate (10.17 g (4.5 mmol) in anhydrous dimethyl sulfoxide (DMSO, 85 mL). The polymer solution was stirred under argon atmosphere for 5 days. Ultrafiltration was performed using a dialysis membrane (molecular weight cut-off = 3500, 1 liter of methanol).

Yield = 6.60 g (61%).

^1^H NMR (CH_3_OD): *δ* 1.24 (m, 2H, CH_2_), 1.52 (bs, 1H, CH), 1.75 (d, 2H, CH_2_), 2.03 (t, 4H, CH_2_), 2.38 (t, 4H, CH_2_), 2.49 (d, 2H, CH_2_), 2.65 (t, 2H, CH_2_), 2.83 (t, 4H, CH_2_), 2.95 (d, 2H, CH_2_), 3.49 (t, 4H, CH_2_), 3.63 (s, b, OCH_2_CH_2_), 4.14 (bs, 2H, CH_2_).

### Synthesis of poly(AMPD-BAC)-*g*-PEG-Biotin-Lipoic acid

Poly(AMPD-BAC)-*g*-PEG (2.0 g, 0.21 mmol) was mixed with NHS-biotin (0.358 g, 1.05 mmol) and alpha-lipoic acid-NHS (0.33 ml, 1.1 mmol) in dry DMSO (30 mL). The mixture was stirred under argon atmosphere at room temperature for 24 h. The resulting solution was washed with water (5 × 500 mL). The solution was then freeze dried to afford poly(AMPD-BAC)-*g*-PEG-biotin-lipoic acid as a light yellow powder. Yield = 1.0 g (50%).

### Synthesis of MMP-2-peptide crosslinked poly(AMPD-BAC)-*g*-PEG-biotin-lipoic acid

MMP-2 peptide (C-G-K-G-P-L-G-V-R-G-C-CONH_2_) was used for crosslinked poly(AMPD-BAC)-*g*-PEG-biotin-lipoic acid. MMP-2-peptide (100 mg, 0.093 mmol) was mixed with acrylic acid N-hydroxysuccinimide ester (100 mg, 0.6 mmol) and triethylamine (0.1 ml, 1 mmol) in dry DMSO (20 mL). The mixture was stirred under argon atmosphere at room temperature for 24 h. Diethyl ether was used to precipitate acrylate MMP-2-peptide. Acrylate MMP-2-peptide (0.1 g, 0.1 mmol) was then mixed with poly(AMPD-BAC)-*g*-PEG-biotin-lipoic acid in DI water (30 mL). The mixture was stirred under argon at 50 °C for 48 h. The resulting solution was washed with water (5 × 500 mL, using dialysis tubing, molecular weight cut-off = 3500). The solution was then freeze dried to afford a light yellow solid.

Yield = 0.90 g (54%).

### Coating of avidin on SERS nanotags

Nanotags (100 μL) was suspended in 1 mL DI water. 1 μL of biotin-PEG_1K_-thiol (50 μM) was added into nanotags solution and incubated for 30 min. The mixture was centrifuged at 177 × g for 1 min to eliminate excess biotin-thiol. The pellet was resuspended in 1 mL DI water and incubated with neutrAvidin (5 mg). After 3 h, the solution was centrifuged at 177 × g for 1 min to eliminate excess neutrAvidin.

### Preparation of SERS nanotags for logical OR and AND sensing

For logical OR and AND sensing, nanotag is protected by MMP-PEG. MMP Peptides were customized and purchased from Shanghai Hanhong Chemical Co., Ltd. (China). An amine-reactive methoxy polyethylene glycol-succimidyl α methylbutanoate (mPEG SMB reagents) was obtained from Nektar Therapeutics. mPEG SMB was conjugated with MMP peptides via the lysine moiety. One milliliter of 1 mM MMP-2 peptide (Gly-Lys-Gly-Pro-Leu-Ala-Nva-Dpa-Ala-Arg-Gly-Cys-CONH_2_), 1 mL of 1 mM MMP-7 peptide (Gly-Lys-Gly-Val-Pro-Leu-Ser-Leu-Thr-Met-Gly-Cys-CONH_2_)^50,65,66^, 1 mL of 1 mM MMP-9 peptide (Gly-Lys-Gly-Pro-Leu-Gly-Leu-Leu-Gly-Cys-CONH_2_), 1 mL of 1 mM MMP-3 peptide (Gly-Lys-Pro-Tyr-Ala-Tyr-Trp-Met-Arg-Cys-CONH_2_), or 1 mL of 1 mM MMP-1 peptide (Pro-Leu-Gly-Cys-His-Ala-D-Arg-NH_2_) was incubated with mPEG SMB in DI water (5 mL) for 24 h. The MMPs-PEG were then incubated with avidin-coated SERS nanotags for 2 h.

### Deposition of nanogel on leaned nanopillars

The deposition process was performed using a fountain pen^67–69^. Fountain pen was from Nanonics (specification: 500–600 µm length, 900 nm aperture diameter and Cr/Au-covered). The nanofountain pen was overflowed with 10 nM of nanogel with a needle. The deposition process was conducted under ambient conditions using an AFM scanning optical microscope system (MV2000 Nanonics). Such a system can precisely position the nanofountain pen on the substrate. The nanofountain pen was moved to the substrate at 300 Hz speed. The drawing process was performed with 0.02 μm/ms and −4.3 imaging set points. After deposition, the substrate was dried in air and kept in a dry box.

### Electromagnetic simulation

A high-mesh-density simulation of nanostructures was conducted by in a Lenovo desktop using a 64-bit Windows 7 Professional operation system, Intel(R) Core(TM)_2_ Quad Q9650 3 GHz with 8 GB of RAM. COMSOL Multiphysics version 5.3 (RF extended module) was used to simulate the plasmonic properties of nanostructures. Prior to the experiment, the exact dimension of nanostructure including boundary conditions and PML (perfectly matched layer) was prepared by Draw Mode with the Cartesian coordinate function. The simulation period for a simple sphere nanostructure was roughly 4 h.

### Detection of MMP-2

Nanogel chip (with MMP-2 crosslinker and biotin) was incubated with MMP-2 enzyme solution for 3 h at 37 °C. The concentration of MMP-2 enzyme (0.05 ng/mL to 50 ng/mL) was varied by dilution using buffer solutions. The buffer solution was prepared by mixing 0.005% Brij-35, 50 mM Tris-Cl, 5 mM CaCl_2_. The pH of the buffer was adjusted to pH 7.4. The substrate was carefully washed with 1 mL of DI water. 10 μL of Os-SH nanotag coated with avidin (without MMP-2-PEG) was added to substrate and incubated for 3 h at 37 °C. The nanogel chip was carefully washed with 1 mL of DI water to eliminate excess nanotags. SERS measurement was then conducted using Raman microscope.

### Detection of MMP-1 and MMP-2 (logical AND detection)

Nanogel chip functionalized with biotin (without MMP-1 and MMP-2 crosslinkers) and W-(CO)_5_ and Os-SH nanotags functionalized with avidin (with MMP-1 or MMP-2-PEG) were used. As such, proteolysis can only occur with the nanotags coated with MMPs-PEG. Nanotags (100 μL of 1 OD) was incubated with the respective MMP enzyme for 3 h at 37 °C. The concentration of MMP enzyme was varied (0.05 ng/mL to 50 ng/mL) by dilution using buffer solutions. The buffer solution was prepared by mixing 0.005% Brij-35, 50 mM Tris-Cl, 5 mM CaCl_2_. The pH of the buffer was adjusted to pH 7.4. After incubation, the nanotags was then loaded to the nanogel chip. After incubation for 3 h, the nanogel substrate was rinsed with 1 mL of DI water to remove unbound nanotags. SERS measurement was then conducted using Raman microscope.

### Detection of MMP-1, MMP-2, MMP-3, MMP-7, and MMP-9 (logical OR detection)

Nanogel chip functionalized with biotin (without MMPs crosslinked) and nanotags functionalized with avidin (with MMP-1-PEG, MMP-2-PEG, MMP-3-PEG, MMP-7-PEG, and MMP-9-PEG) were used. Therefore, proteolysis can occur with nanotags. Nanotags (100 μL of 1 OD) were incubated with the respective MMP enzymes solution at 37 °C for 3 h. The concentration of MMP enzymes was varied (0.05 ng/mL to 50 ng/mL) by dilution using buffer solutions. The buffer solution was prepared by mixing 0.005% Brij-35, 50 mM Tris-Cl, 5 mM CaCl_2_. The pH of the buffer was adjusted to pH 7.4. After incubation, the nanotags was then loaded to the nanogel chip. After incubation for 3 h, the nanogel substrate was rinsed with 1 mL of DI water to remove unbound nanotags. SERS measurement was then conducted using Raman microscope.

### Estimation of enhancement factor for Os-CO nanotag

The SERS enhancement was estimated by the intensity of carbonyl peak from nanotags and carbonyl peak from metal carbonyl compounds as:

1 $$\,{\rm{Enhancement}}\,{\rm{factor}}=({C}_{{\rm{metal}}{\rm{carbonyl}}}\times {I}_{{\rm{nanotags}}})\div({C}_{{\rm{nanotags}}}\times {I}_{{\rm{metal}}{\rm{carbonyl}}})$$

where *C*_metal carbonyl_ and *C*_nanotags_ are the concentration and *I*_metal carbonyl_ and *I*_nanotags_ are the corresponding normal Raman and SERS intensities for the metal carbonyl and the nanotags, respectively. For the carbonyl peak associated with metal carbonyl compounds (normal Raman peak), the volume of metal carbonyl was estimated based on the diameter of focused laser (a cylinder shape with diameter = 25 μm) and height of focus depth (height of 1 μm). The amount of Os-SH in nanoparticles was estimated by measuring the concentration of osmium element left behind in the preparation of Os-SH nantags using inductively coupled plasma mass spectrometry (ICP-MS). Thus, the amount of Os-SH on gold nanoparticles can be estimated by using the original amount of Os-SH to substrate the unreacted amount. The amount of Os-SH was divided by the concentration of gold nanoparticles. The concentration of gold nanoparticles can be measured by its optical density (BBInternational UK product data sheet). The concentration of metal carbonyl compounds per gold nanoparticle was found to be 1632 ± 142. Using the above information, the concentration of Os-SH on nanoparticles used for SERS was 1.81 nmol to generate 1200 intensity counts, and 50 mM Os-SH was used for Raman measurements to generate ~800 Raman intensity counts. The enhancement factor can be estimated by substituting these values into the Eq. (1) to obtain 6.6 × 10^7^.

### Study approval and clinical blood samples collection

This clinical study was approved by the Ethics Committee of Fujian Cancer Hospital (SQ2018-010-01). An informed written consent was obtained from all participants. A total of 30 blood plasma samples were collected in this study, including 3 samples from NPC patients with stage I, 7 samples from NPC patients with stage II, 10 samples from NPC patients with stage III and 10 samples from NPC patients with stage IV as determined by standard histopathological diagnosis. More clinical information on these patients could be obtained from Supplementary Table 2. It should be noted that the concentration of Epstein-Barr virus DNA was detected by using a commercial kit (DAAN GENE) combined with a real-time fluorescence quantitative PCR system (Applied Biosystems 7500). The primers used for Epstein-Barr virus DNA detection in PCR are shown in Supplementary Table 6. The blood plasma samples of NPC patients were provided by the Fujian Cancer Hospital, China. After 12 h of overnight fasting, peripheral blood (5 mL) was collected from each subject. The blood was transferred into an EDTA tube for the isolation of plasma between 7:00–8:00 AM. The samples were then stored at −80 °C until further use. All patient sample experiments were done in a blinded manner. All patient samples had been de-identified by a research assistant who is not involved in the study. The results were only revealed after the completion of SERS experiments.

### SERS experiments for clinical blood sample

For blood sample testing, 5 μL of blood plasma sample was introduced to nanotags (100 μL of 1 OD) in an Eppendorf tube containing 10 μL of buffer solutions. The buffer solution was prepared by mixing 0.005% Brij-35, 50 mM Tris-Cl, 5 mM CaCl_2_. The pH of the buffer was adjusted to pH 7.4. The tube was vortex-mixed for 30 s. The solution was transferred to a nanogel chip functionalized with biotin (without MMPs-PEG crosslinkers) and incubated at 37 °C for 3 h. After that, the nanogel chip was rinsed with 1 mL of DI water. SERS measurement was then conducted using Raman microscope.

Furthermore, the detection deviation of SERS method and ELISA method is calculated:

2 $$[({C}_{{\rm{SERS}}}-{C}_{{\rm{ELISA}}})\div{C}_{{\rm{ELISA}}}]\times 100 \%$$

where C_SERS_ and C_ELISA_ represent the concentration of the MMP obtained by SERS and ELISA assay, respectively.

### Enzyme-linked immunosorbent assay (ELISA)

The concentrations of MMP-1, MMP-2, MMP-3, MMP-7 and MMP-9 in human plasma samples were measured using commercial ELISA kits (Elabscience). The assays were assessed according to the instructions provided by manufacturer. 100 μL of standards and blood plasma samples were added into ELISA plates. The plate was incubated at 37 °C for 90 min. After that, the ELISA wells were carefully washed with washing buffer (5 × 100 μL). Then, a 100 μL of biotinylated detection antibody was added into wells and incubated for 60 min at 37 °C. The wells were subsequently washed five times with washing buffer. Then, HRP conjugate working solution (100 μL) was introduced into wells and incubated for 30 min at 37 °C. The reaction wells were carefully rinsed with washing buffer (5 × 100 μL), followed by a 15 min incubation with substrate reagent (100 μL) at 37 °C. Prior to the optical measurement, a stop solution (50 μL) was introduced into reaction wells. The absorbance for each well at 450 nm was measured (CMax Plus, Molecular Devices). The data analysis was performed using SoftMax Pro software version 7 (Molecular Devices).

### Statistical analysis

Statistical analyses were performed using the Sangerbox tools, an online platform for data analysis (http://www.sangerbox.com/tool). The line within each notch box represents the median, and the lower and upper boundaries of the box indicate the first and third quartiles, respectively (Fig. 8). The *whiskers* represent the 1.5x interquartile range. *P*-values < 0.05 were considered statistically significant. The exact *P*-values for each combination are summarized in Supplementary Table 4. The correction coefficient (*r*) was obtained by performing the Pearson’s correction analysis (Fig. 9). The histograms in Fig. 9b–f represent the frequency distribution of MMPs and EBV levels. Two-sided Student’s *t*-test was used for statistical analysis, and no adjustments were made for multiple comparisons.

### Reporting summary

Further information on research design is available in the Nature Research Reporting Summary linked to this article.

## Supplementary information

Supplementary Information

Reporting Summary

## Data Availability

Data are available within the article and supplementary files. The source data underlying Figs. 2c, d, e, f, 5d, f, 6a and b are provided as a Source Data file. All other data that support the findings of the study are available from the corresponding author upon reasonable request. Source data are provided with this paper.

## Source data

Source Data

## Code availability

The software used for statistical analyses is available at http://www.sangerbox.com/tool.

## References

[1] Kao Y-C (2020). Multiplex surface-enhanced Raman scattering identification and quantification of urine metabolites in patient samples within 30 min. ACS Nano.

[2] Zhang Y (2018). Classifying low-grade and high-grade bladder cancer using label-free serum surface-enhanced Raman spectroscopy and support vector machine. Laser Phys..

[3] Quintero-Fabián S (2019). Role of matrix metalloproteinases in angiogenesis and cancer. Front. Oncol..

[4] Page-McCaw A, Ewald AJ, Werb Z (2007). Matrix metalloproteinases and the regulation of tissue remodelling. Nat. Rev. Mol. Cell Biol..

[5] Pittayapruek P, Meephansan J, Prapapan O, Komine M, Ohtsuki M (2016). Role of matrix metalloproteinases in photoaging and photocarcinogenesis. Int. J. Mol. Sci..

[6] Gonzalez-Avila G (2019). Matrix metalloproteinases participation in the metastatic process and their diagnostic and therapeutic applications in cancer. Crit. Rev. Oncol. /Hematol..

[7] Gong T, Kong KV, Goh D, Olivo M, Yong K-T (2015). Sensitive surface enhanced Raman scattering multiplexed detection of matrix metalloproteinase 2 and 7 cancer markers. Biomed. Opt. Express.

[8] Gong T (2017). Optical interference-free surface-enhanced Raman scattering CO-nanotags for logical multiplex detection of vascular disease-related biomarkers. ACS Nano.

[9] Granger JH, Granger MC, Firpo MA, Mulvihill SJ, Porter MD (2013). Toward development of a surface-enhanced Raman scattering (SERS)-based cancer diagnostic immunoassay panel. Analyst.

[10] Gao H (2017). Highly selective electrogenerated chemiluminescence biosensor for simultaneous detection of matrix metalloproteinase-2 and matrix metalloproteinase-7 in cell secretions. Sens. Actuators B Chem..

[11] Tan MJ (2017). Metal carbonyl-gold nanoparticle conjugates for highly sensitive SERS detection of organophosphorus pesticides. Biosens. Bioelectron..

[12] Nemecek, D., Stepanek, J., Thomas, G. J. Raman spectroscopy of proteins and nucleoproteins. In: *Current Protocols in Protein Science* (ed Coligan J. E.). (John Wiley & Sons, Inc. Press, 2013). 10.1002/0471140864.ps1708s71 23377849

[13] Kong KV, Lam Z, Goh WD, Leong WK, Olivo M (2012). Metal carbonyl–gold nanoparticle conjugates for live-cell SERS imaging. Angew. Chem. Int. Ed..

[14] Kong KV, Dinish US, Lau WKO, Olivo M (2014). Sensitive SERS-pH sensing in biological media using metal carbonyl functionalized planar substrates. Biosens. Bioelectron..

[15] Kong KV, Lam Z, Lau WKO, Leong WK, Olivo M (2013). A transition metal carbonyl probe for use in a highly specific and sensitive SERS-based assay for glucose. J. Am. Chem. Soc..

[16] Garimella SV, Drozd V, Durygin A (2009). High-pressure Raman study on the decomposition of polycrystalline molybdenum hexacarbonyl. J. Inorg. Organomet. Polym. Mater..

[17] Garimella S, Drozd V, Durygin A, Chen J (2012). High pressure Raman and x-ray diffraction studies on the decomposition of tungsten carbonyl. J. Appl. Phys..

[18] Burdett JK (1978). Characterization by infrared and Raman spectroscopy of matrix-isolated M(CO)_5_N_2_ (M = chromium, molybdenum, or tungsten) produced by photolysis of M(CO)_6_. Inorg. Chem..

[19] Morichika I, Murata K, Sakurai A, Ishii K, Ashihara S (2019). Molecular ground-state dissociation in the condensed phase employing plasmonic field enhancement of chirped mid-infrared pulses. Nat. Commun..

[20] Ahmed MOE, Leong WK (2006). Colloidal silver nanoparticles stabilized by a water-soluble triosmium cluster. J. Organomet. Chem..

[21] El-Sayed MA, Kaesz HD (1962). Assignment of the CO stretching absorptions in C_4v_ metal pentacarbonyl derivatives. The method of local oscillating dipoles. J. Mol. Spectrosc..

[22] Bistoni G (2016). How π back-donation quantitatively controls the CO stretching response in classical and non-classical metal carbonyl complexes. Chem. Sci..

[23] Patmore NJ, Steed JW, Weller AS (2000). Transition metal complexes of the weakly coordinating carborane anion [CB_11_H_12_]-: the first isolation and structural characterisation of an intermediate in a silver salt metathesis reaction. Chem. Commun..

[24] Sakthivel A, Zhao J, Raudaschl-Sieber G, Kühn FE (2005). In situ grafting of cyclopentadienyl-molybdenum complexes on mesoporous materials: The reaction of [η^5^-CpMo(CO)_3_]−Na^+^ with surface fixed iodo-benzyl siloxane. J. Organomet. Chem..

[25] Otten MM, Lamb HH (1994). C- and O-bonded carbon monoxide on surfaces: interactions of [CpW(CO)_3_]- with cations on magnesia, alumina, and potassium-modified alumina. J. Am. Chem. Soc..

[26] Bitner TW, Wedge TJ, Hawthorne MF, Zink JI (2001). Synthesis and luminescence spectroscopy of a series of [η^5^-CpFe(CO)_2_] complexes containing 1,12-Dicarba-closo-dodecaboranyl and -ylene ligands. Inorg. Chem..

[27] Lin Y-C, Ke Z-Y, Liao P-H, Tseng C-Y, Kong KV (2020). Reversible detection of phosphorylation and dephosphorylation by tip-enhanced Raman spectroscopy using a cyclopentadienyl ruthenium nanotag functionalized tip. Chem. Commun..

[28] Schilling BER, Hoffmann R, Lichtenberger DL (1979). CpM(CO)_2_(ligand) (Cp = cyclopentadienyl, M = metal) complexes. J. Am. Chem. Soc..

[29] Gregory MF, Poliakoff M, Turner JJ (1985). Infrared spectra of 13CO-enriched Ru(CO)_5_ in liquid xenon: The energy-factored force field. J. Mol. Struct..

[30] Ishikawa Y, Kawakami K (2007). Structure and infrared spectroscopy of group 6 transition-metal carbonyls in the gas phase: DFT studies on M(CO)_n_ (M = Cr, Mo, and W; n = 6, 5, 4, and 3). J. Phys. Chem. A.

[31] Chevreau H, Martinsky C, Sevin A, Minot C, Silvi B (2003). The nature of the chemical bonding in the D_3h_ and C_2v_ isomers of Fe_3_(CO)12. N. J. Chem..

[32] Huggins DK, Flitcroft N, Kaesz HD (1965). Infrared spectrum of osmium tetracarbonyl trimer, Os_3_(CO)_12_; assignment of CO and MC stretching absorptions. Inorg. Chem..

[33] Quicksall CO, Spiro TG (1968). Raman frequencies of metal cluster compounds: triosmium dodecacarbonyl and triruthenium dodecacarbonyl. Inorg. Chem..

[34] Monari M, Pfeiffer R, Rudsander U, Nordlander E (1996). Synthesis of new thiol derivatives of [Os_3_(CO)_12_]; crystal structure of [Os_3_(CO)_10_(μ-H){μ-SC)(CH_3_)_3_}]. Inorg. Chim. Acta.

[35] Sharpe, C. H. A. A. G. Inorganic Chemistry (5th Edition). (Pearson Education, Press, Harlow, 2018).

[36] Slebodnick C (2004). High pressure study of Ru_3_(CO)_12_ by X-ray diffraction, Raman, and infrared spectroscopy. Inorg. Chem..

[37] Lum MW, Leong WK (2001). Chemical transformations on diols anchored to a triosmium cluster. J. Chem. Soc., Dalton Trans..

[38] Ainscough EW, Brodie AM, Coll RK, Coombridge BA, Waters JM (1998). The reaction of [Os_3_(CO)_10_(CH_3_CN)_2_] with carboxylic acids: the crystal and molecular structures of [Os_3_H(CO)_10_(C_6_H_4_(OH)CO_2_)] and [Os_3_H(CO)10(C_6_H_5_COS)]. J. Organomet. Chem..

[39] Schilling BER, Hoffmann R (1979). M3L9(ligand) complexes. J. Am. Chem. Soc..

[40] Gazquez JL, Mendez F (1994). The hard and soft acids and bases principle: an atoms in molecules viewpoint. J. Phys. Chem..

[41] Pearson RG, Songstad J (1967). Application of the principle of hard and soft acids and bases to organic chemistry. J. Am. Chem. Soc..

[42] Xu H, Xu DC, Wang Y (2017). Natural indices for the chemical hardness/softness of metal cations and ligands. ACS Omega.

[43] Pyper KJ (2013). Microwave promoted oxidative addition reactions of Os_3_(CO)_12_: efficient syntheses of triosmium clusters of the type Os_3_(μ-X)_2_(CO)10 and Os_3_(μ-H)(μ-OR)(CO)_10_. J. Clust. Sci..

[44] Chan KH (2006). Organometallic cluster analogues of tamoxifen: synthesis and biochemical assay. J. Organomet. Chem..

[45] Dalirsani Z (2019). Comparison of matrix metalloproteinases 2 and 9 levels in saliva and serum of patients with head and neck squamous cell carcinoma and healthy subjects. Int J. Cancer Manag.

[46] Görögh T (2006). Metalloproteinases and their inhibitors: influence on tumor invasiveness and metastasis formation in head and neck squamous cell carcinomas. Head. Neck.

[47] Li S, Luo W (2019). Matrix metalloproteinase 2 contributes to aggressive phenotype, epithelial-mesenchymal transition and poor outcome in nasopharyngeal carcinoma. Onco. Targets Ther..

[48] Yang R (2013). Combined upregulation of matrix metalloproteinase-1 and proteinase-activated receptor-1 predicts unfavorable prognosis in human nasopharyngeal carcinoma. Onco. Targets Ther..

[49] Xia X (2012). Quantifying the coverage density of poly(ethylene glycol) chains on the surface of gold nanostructures. ACS Nano.

[50] von Maltzahn G (2007). Nanoparticle self-assembly gated by logical proteolytic triggers. J. Am. Chem. Soc..

[51] Littlepage LE (2010). Matrix metalloproteinases contribute distinct roles in neuroendocrine prostate carcinogenesis, metastasis, and angiogenesis progression. Cancer Res..

[52] Shuman Moss LA, Jensen-Taubman S, Stetler-Stevenson WG (2012). Matrix metalloproteinases: changing roles in tumor progression and metastasis. Am. J. Pathol..

[53] Andrea Page-McCaw AJE (2007). Zena Werb. Matrix metalloproteinases and the regulation of tissue remodelling. Nat. Rev. Mol..

[54] Roy R, Yang J, Moses MA (2009). Matrix metalloproteinases as novel biomarkers and potential therapeutic targets in human cancer. J. Clin. Oncol..

[55] Kondo S (2005). Epstein-Barr virus latent membrane protein 1 induces the matrix metalloproteinase-1 promoter via an Ets binding site formed by a single nucleotide polymorphism: enhanced susceptibility to nasopharyngeal carcinoma. Int. J. Cancer.

[56] Nasr HB, Chahed K, Remadi S, Zakhama A, Chouchane L (2009). Expression and clinical significance of latent membrane protein-1, matrix metalloproteinase-1 and Ets-1 transcription factor in Tunisian nasopharyngeal carcinoma patients. Arch. Med. Res..

[57] Li Y, Feng Z, Xing S, Liu W, Zhang G (2020). Combination of serum matrix metalloproteinase-3 activity and EBV antibodies improves the diagnostic performance of nasopharyngeal carcinoma. J. Cancer.

[58] Maurel J (2010). Serum matrix metalloproteinase 7 levels identifies poor prognosis advanced colorectal cancer patients. Int. J. Cancer.

[59] Liu Z (2010). Increased expression of MMP9 is correlated with poor prognosis of nasopharyngeal carcinoma. BMC Cancer.

[60] Chan KCA (2014). Plasma Epstein-Barr virus DNA as a biomarker for nasopharyngeal carcinoma. J. Cancer.

[61] Chen Y-P (2019). Nasopharyngeal carcinoma. Lancet.

[62] Lu J, Chua HH, Chen SY, Chen JY, Tsai CH (2003). Regulation of matrix metalloproteinase-1 by Epstein-Barr virus proteins. Cancer Res.

[63] Kuppers DA, Lan K, Knight JS, Robertson ES (2005). Regulation of matrix metalloproteinase 9 expression by Epstein-Barr virus nuclear antigen 3C and the suppressor of metastasis Nm23-H1. J. Virol..

[64] Serra D, Correia MC, McElwee-White L (2011). Iron and ruthenium heterobimetallic carbonyl complexes as electrocatalysts for alcohol oxidation: electrochemical and mechanistic studies. Organometallics.

[65] Yoon SM (2011). Application of near-infrared fluorescence imaging using a polymeric nanoparticle-based probe for the diagnosis and therapeutic monitoring of colon cancer. Dig. Dis. Sci..

[66] Xu J (2019). A peptide-based four-color fluorescent polydopamine nanoprobe for multiplexed sensing and imaging of proteases in living cells. Chem. Commun..

[67] Gordon MR (2018). Matrix metalloproteinase-9-responsive nanogels for proximal surface conversion and activated cellular uptake. Biomacromolecules.

[68] Kantarovich K, Tsarfati I, Gheber LA, Haupt K, Bar I (2009). Writing droplets of molecularly imprinted polymers by nano fountain pen and detecting their molecular interactions by surface-enhanced Raman scattering. Anal. Chem..

[69] Belmont A-S, Sokuler M, Haupt K, Gheber LA (2007). Direct writing of molecularly imprinted microstructures using a nanofountain pen. Appl. Phys. Lett..

