# Supplementary material for: Geometrically encoded SERS nanobarcodes for the logical detection of nasopharyngeal carcinoma-related progression biomarkers
Source: Nat Commun. 2021 Jun 8;12:3430. doi: 10.1038/s41467-021-23789-3 (PMC8173014; doi:10.1038/s41467-021-23789-3)
Supplement: Supplementary file 1 — Supplementary Information [file 41467_2021_23789_MOESM1_ESM.pdf]

Supplementary Information

Geometrically Encoded SERS Nanobarcodes for the Logical Detection of Nasopharyngeal Carcinoma-related Progression Biomarkers

Duo Lin[1,7], Chang-Lin Hsieh[2,7], Keng-Jia Hsu[2], Pei-Hsuan Liao[2], Sufang Qiu[3], Tianxun Gong[4], Ken-Tye Yong[5,6], Shangyuan Feng[1] and Kien Voon Kong[2*]

[1]Key Laboratory of OptoElectronic Science and Technology for Medicine, Ministry of Education, Fujian Provincial Key Laboratory for Photonics Technology, Fujian Normal University, Fuzhou, Fujian 350007, China.

[2]Department of Chemistry, National Taiwan University, Taipei, 10617, Taiwan.

[3] Fujian Medical University Cancer Hospital, Fujian Cancer Hospital, Fuzhou, Fujian 350014, China.

[4]State Key Laboratory of Electronic Thin Films and Integrated Devices, School of Electronic Science and Engineering (National Exemplary School of Microelectronics), University of Electronic Science and Technology of China, Chengdu, 610054 China.

[5]School of Biomedical Engineering, The University of Sydney, New South Wales 2006, Australia.

[6]The University of Sydney Nano Institute, The University of Sydney, Sydney, New South Wales 2006, Australia.

[7]These authors contributed equally to this work: Duo Lin, Chang-Lin Hsieh.

[*]Corresponding author: K. V. Kong. Email: kvkong@ntu.edu.tw

Contents

Supplementary Figures

Supplementary Figure 1. Narrow line width analysis for the totally symmetric mode for $W(CO)_5$-Au.

Supplementary Figure 2. Proposed interaction of the metal carbonyl with Au NPs.

Supplementary Figure 3. EDX of $CpOs(CO)_2$ moiety on gold nanoparticles.

Supplementary Figure 4. IR spectrum of $CpOs(CO)_2I$.

Supplementary Figure 5. IR spectrum of $CpRu(CO)_2I$.

Supplementary Figure 6. IR spectrum of $CpRu(^{13}CO)_2I$.

Supplementary Figure 7. Solid IR and Raman spectra of $Os_3(CO)_{12}$

Supplementary Figure 8. IR spectrum of Os_3-SH ($Os_3(CO)_{10}(\mu$-$S(CH_2)_8SH)$).

Supplementary Figure 9. NMR spectrum of Os_3-SH ($Os_3(CO)_{10}(\mu$-$S(CH_2)_8SH)$). The linkage of thiol will exhibit a low-field peak at -17 ppm.

Supplementary Figure 10. Character table of Cs point group.

Supplementary Figure 11. ToF-SIMS spectrum (negative ion mode) of Os_3-SH nanotags.

Supplementary Figure 12. IR spectrum of Os_3-COOH nanotag.

Supplementary Figure 13. ToF-SIMS spectrum (negative ion mode) of Os_3-COOH nanotags.

Supplementary Figure 14. IR spectrum of Os_3-OH nanotags.

Supplementary Figure 15. ToF-SIMS spectrum (negative ion mode) of Os_3-OH nanotags.

Supplementary Figure 16. Partial MO diagram for $M_3(CO)_{12}$.

Supplementary Figure 17. Full range Raman spectra for all 14 nanotags.

Supplementary Figure 18. TEM of the nanotags (Os_3-SH nanotag).

Supplementary Figure 19. Leaned nanopillars analysis.

Supplementary Figure 20. NMR spectra of poly(BAC-AMPD)s before and after conjugation of biotin and lipoic acid and crosslinking with the MMP-2 peptide.

Supplementary Figure 21. Deposition of nanogel using fountain pen.

Supplementary Figure 22. SEM image of fountain pen with a small aperture.

Supplementary Figure 23. Hydrodynamic diameter and zeta potential of nanotags.

Supplementary Figure 24. Signals of nanotags observed over 6 months.

Supplementary Figure 25. PEG-peptide SERS spectrum.

Supplementary Figure 26. Schematic representation of unmixed colocalization of five mixtures of MMPs.

Supplementary Figure 27. The SERS detection for (a-b) MMP-7, (c-d) MMP-9, (e-f) MMP-1, (g-h) MMP-2, (i-j) MMP-3.

Supplementary Figure 28. Preparation of SERS nanotags.

Supplementary Tables

Supplementary Table 1. The coefficient of variation (CV) for SERS sensors.

Supplementary Table 2. Patient characteristics

Supplementary Table 3. Concentration of MMPs in clinical blood samples measured by SERS and ELISAs.

Supplementary Table 4. The *P* values for the correlation between the clinical pathological parameters and MMPs level detected by the SERS sensor.

Supplementary Table 5. Comparison between the SERS detection method and other analytic methods.

Supplementary Table 6. The primers used for EBV-DNA detection in PCR

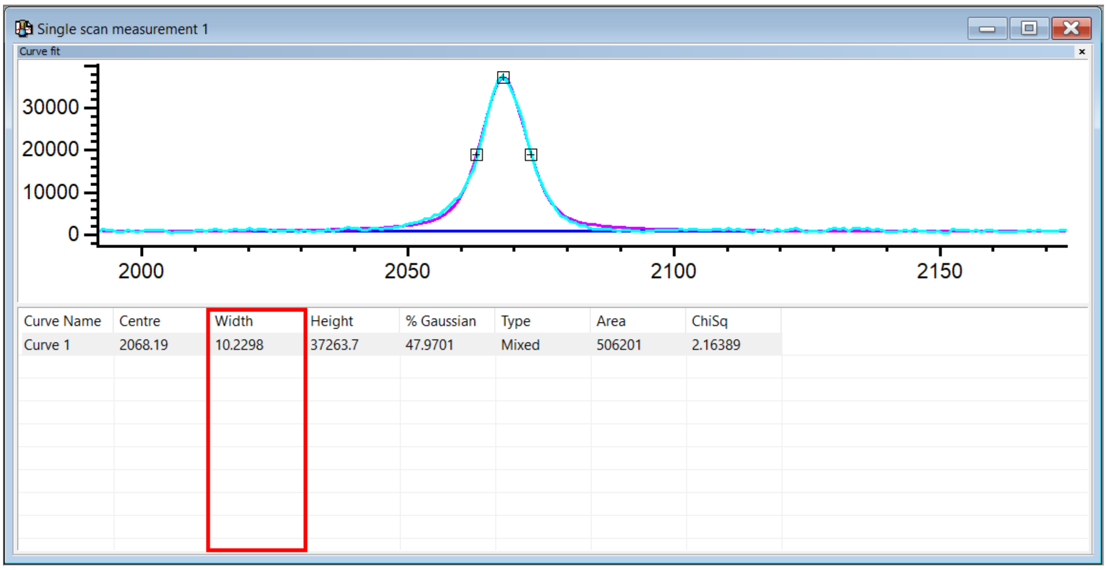

Supplementary Figure 1. Narrow line-width analysis for the totally symmetric mode for $W(CO)_5$-Au.

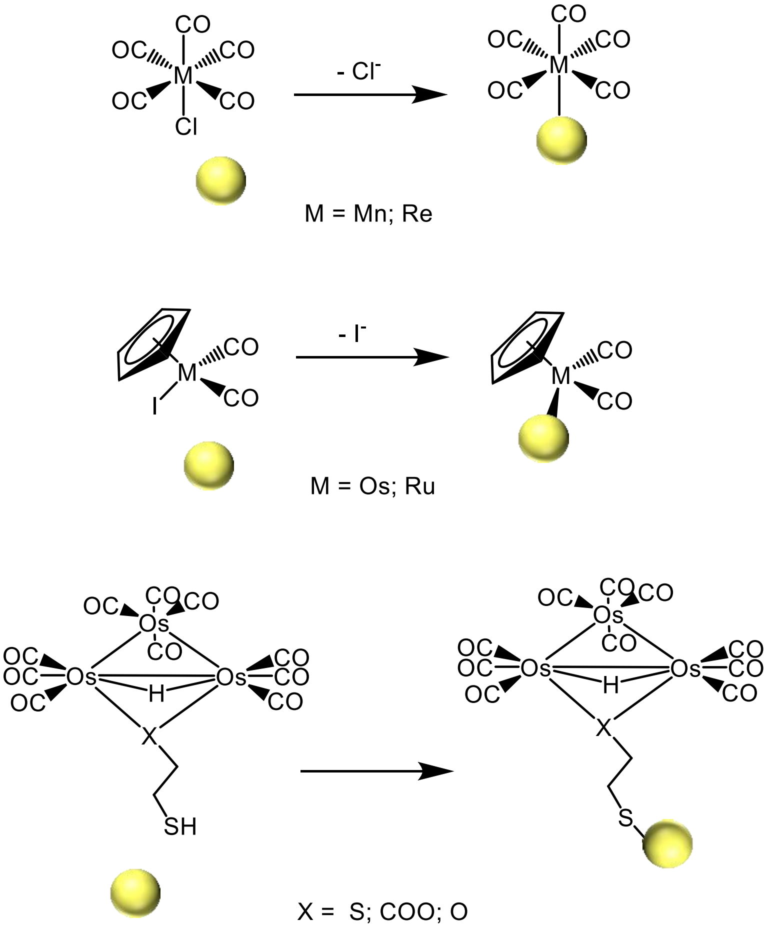

Supplementary Figure 2. Proposed interaction of the metal carbonyl with Au NPs. Thiolated Os_3 with a carbon linker ($HS(CH_2)_8SH$, $HO(CH_2)_{10}SH$ and $HOOC(CH_2)_{10}SH$) and a free -SH

functionality. The Os_3 was immobilized onto Au NP via -SH group. -SH is needed to anchor the clusters onto the Au NP surface via Au-S bonds. The metal hexacarbonyl and cyclopentadienyl metal carbonyl complexes, which contain an iodo or chloro functionality, can directly interact between the Au NP and the metal moiety with the elimination of iodide or chlorine, as the leaving group was deduced to be the mode of interaction.

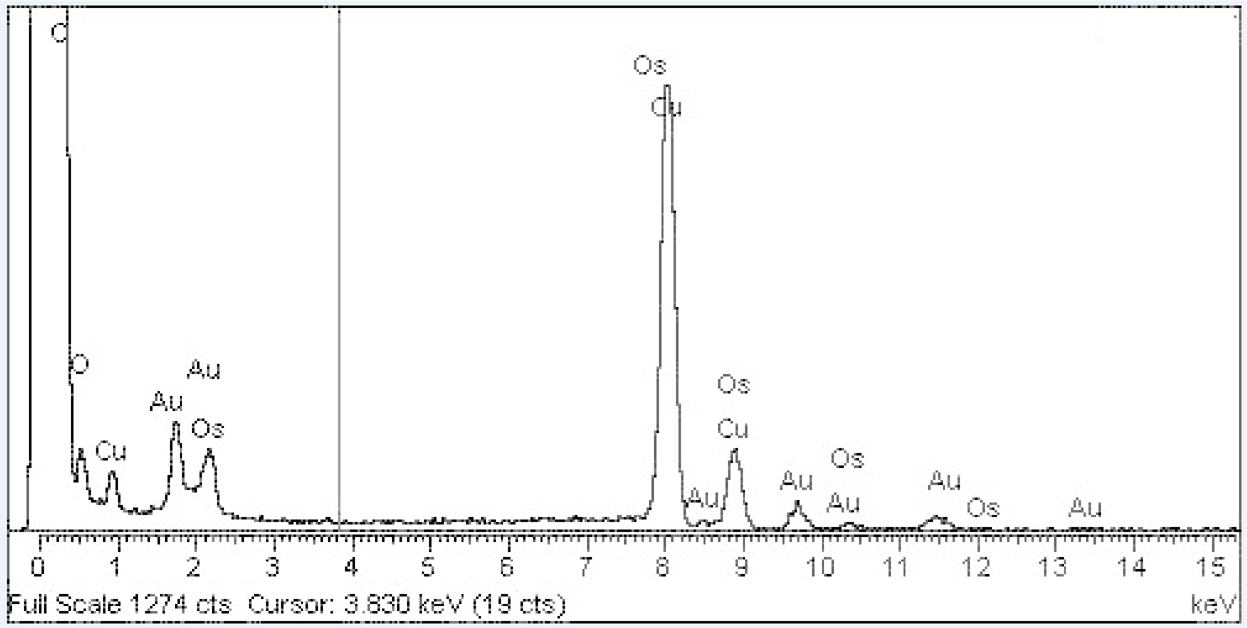

Supplementary Figure 3. EDX of $CpOs(CO)_2$ moiety on gold nanoparticles.

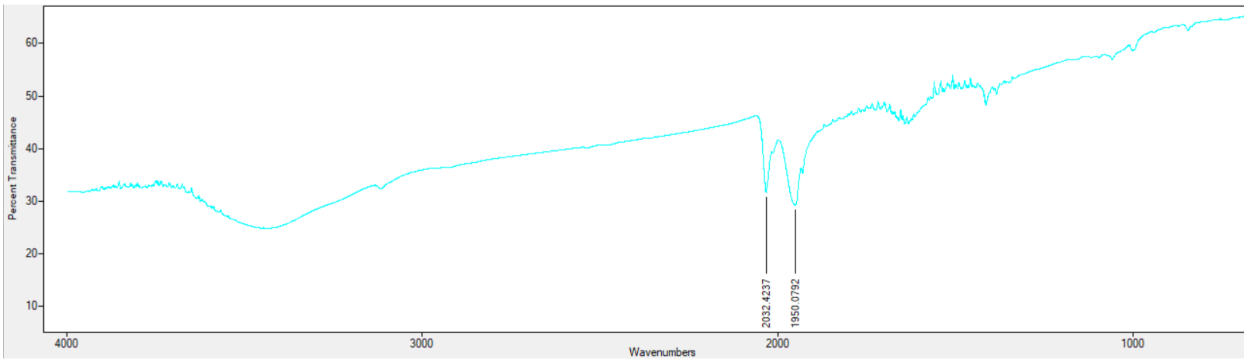

Supplementary Figure 4. IR spectrum of $CpOs(CO)_2I$.

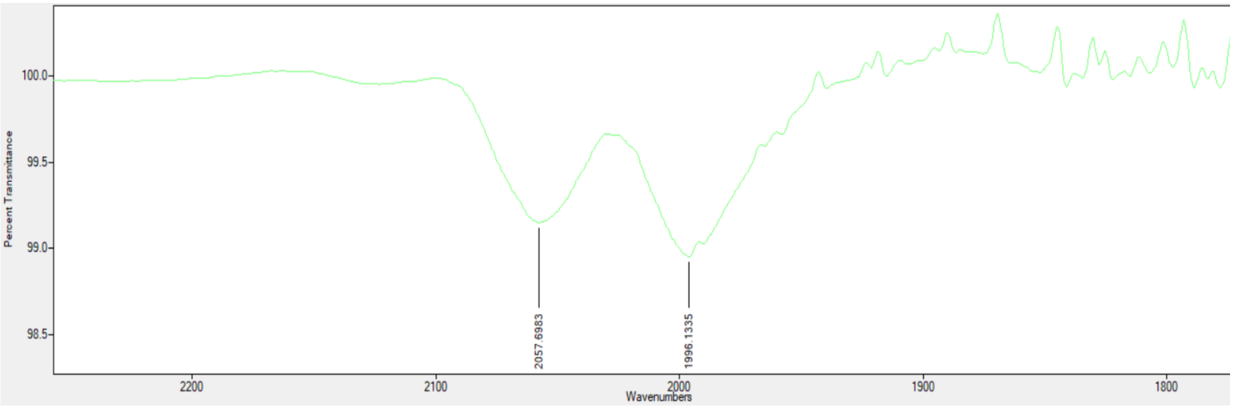

Supplementary Figure 5. IR spectrum of $CpRu(CO)_2I$.

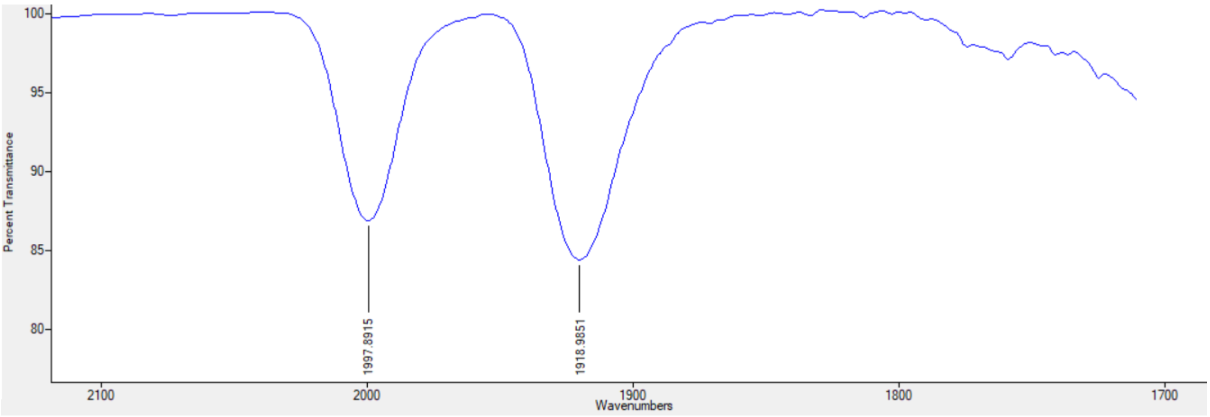

Supplementary Figure 6. IR spectrum of $CpRu(^{13}CO)_2I$.

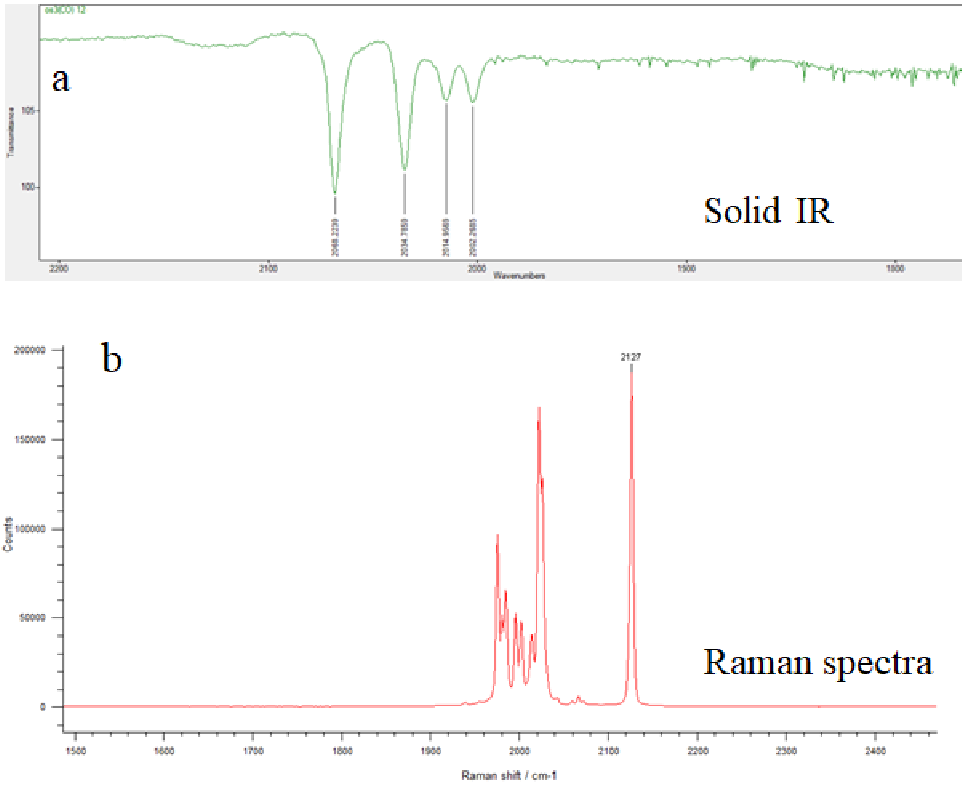

Supplementary Figure 7. (a) Solid IR and (b) Raman spectra of $Os_3(CO)_{12}$

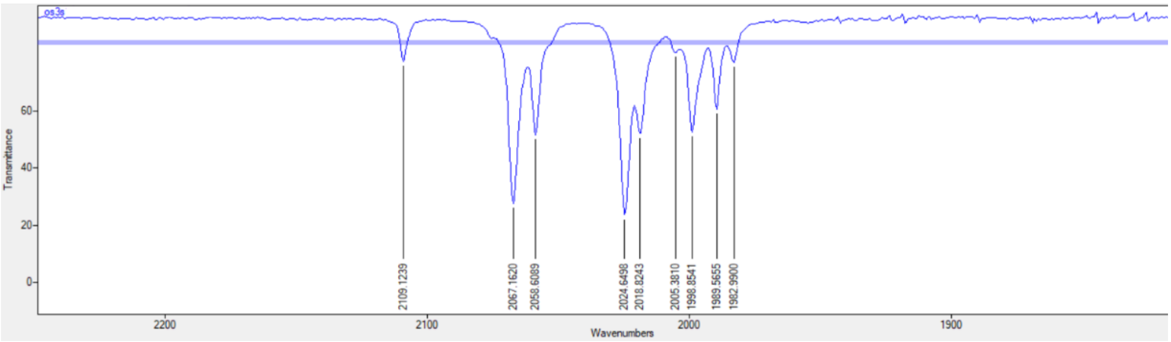

Supplementary Figure 8. IR spectrum of Os_3-SH ($Os_3(CO)_{10}(\mu$-$S(CH_2)_8SH)$).

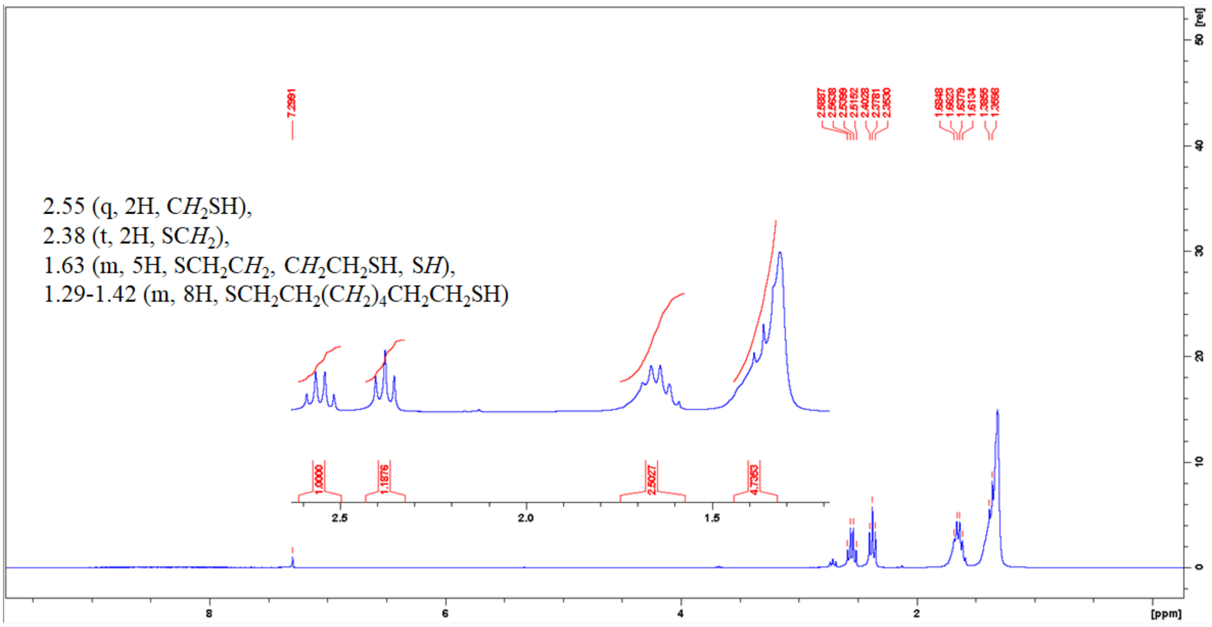

Supplementary Figure 9. NMR spectrum of Os3-SH ($Os_3(CO)_{10}$(μ-S(CH_2)$_8$SH)). The linkage of thiol will exhibit a low-field peak at -17 ppm.

Cs	E	σ_h		
A′	1	1	x, y, R_z	X^2, y^2, Z^2, xy
A″	1	-1	Z, R_x, R_y	yz, xz

symmetry has a quadratic function (i.e, xy or x_2) will be Raman active

Supplementary Figure 10. Character table of the Cs point group.[1]

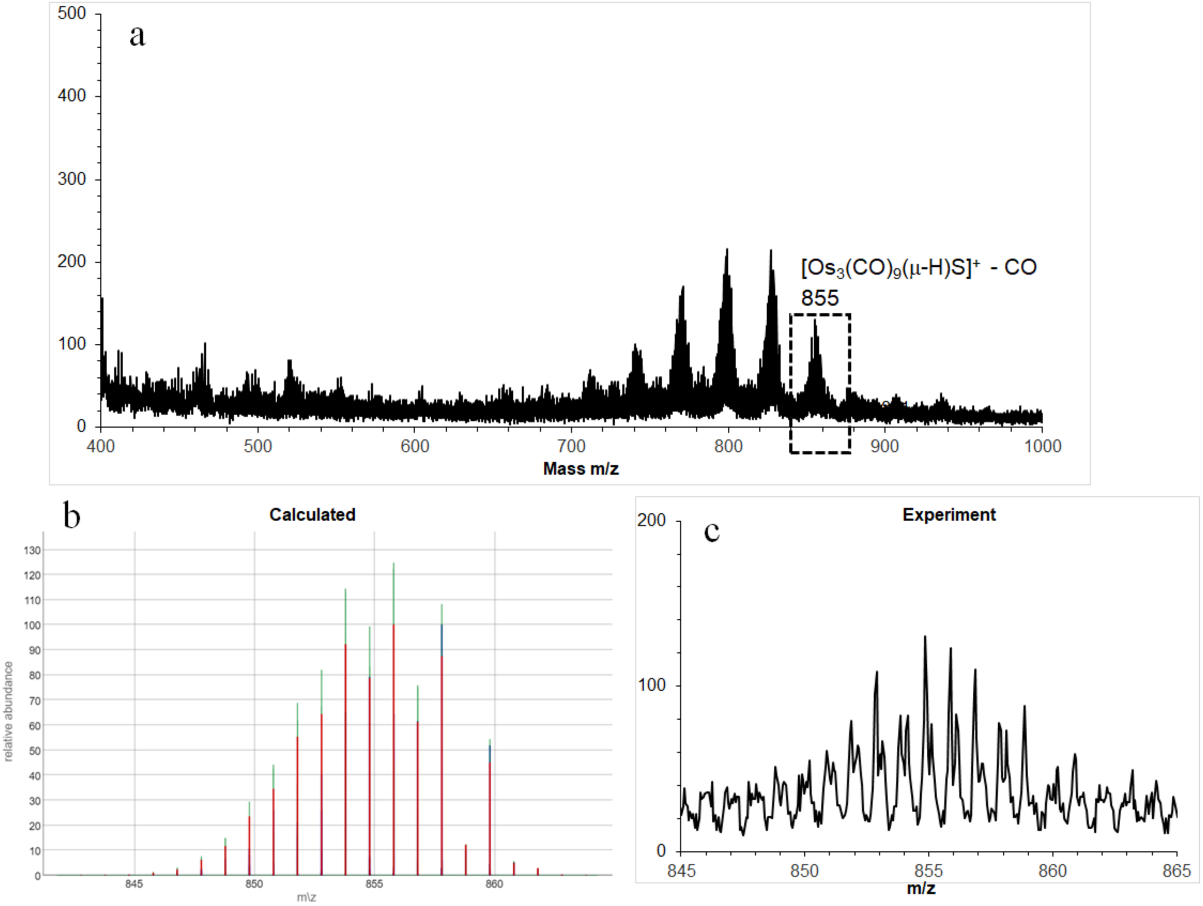

Supplementary Figure 11. ToF-SIMS spectrum (negative ion mode) of Os_3-SH nanotags. (a) Full spectrum of ToF-SIMS of Os_3-SH nanotags. (b) Calculated isotope distribution. (c) Isotope distribution obtained from experiment.

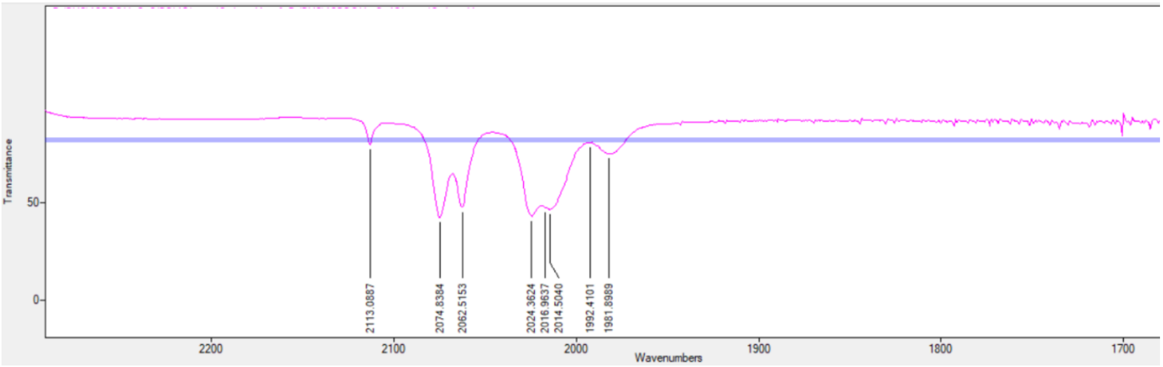

Supplementary Figure 12. IR spectrum of the Os_3-COOH nanotag.

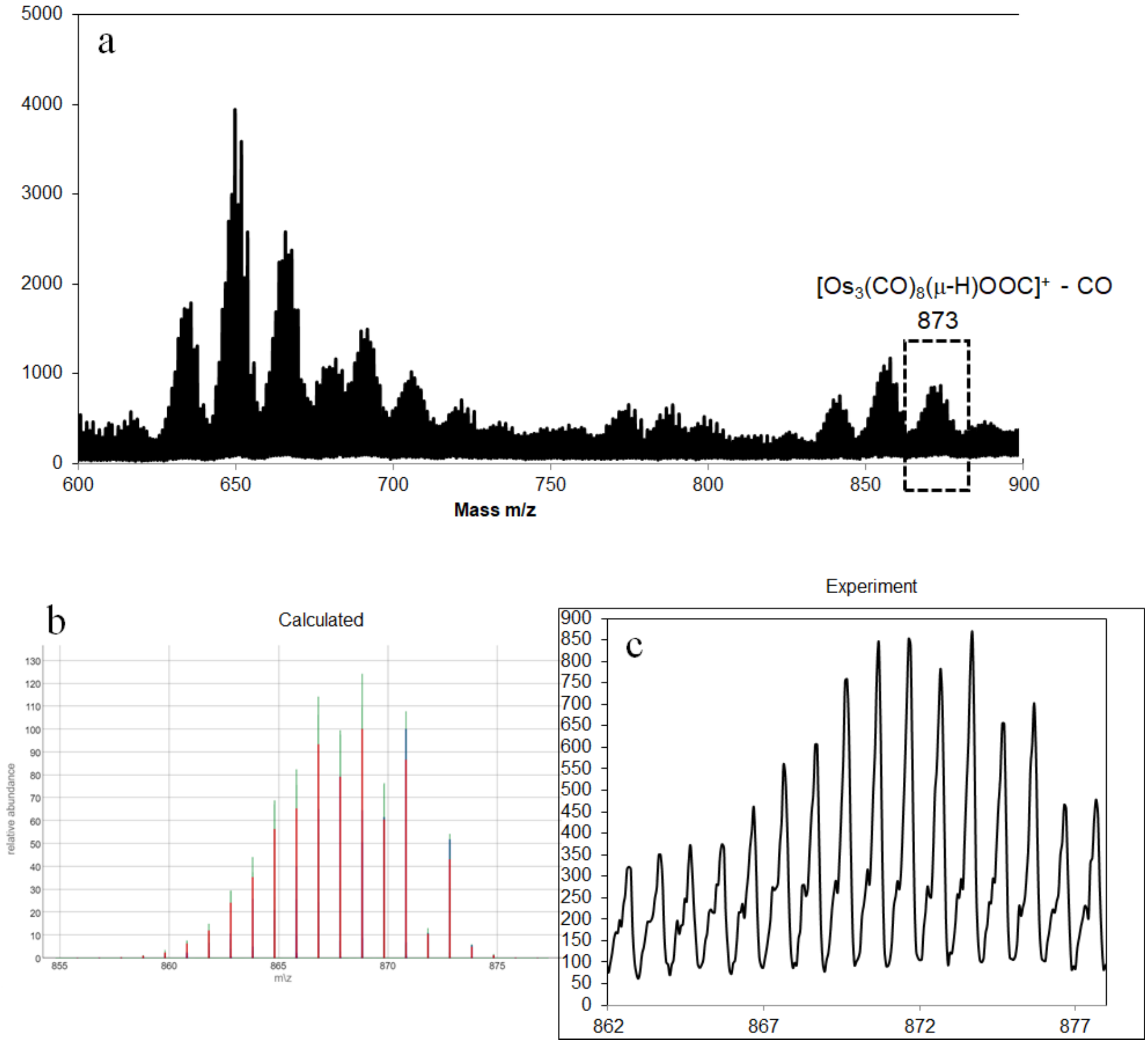

Supplementary Figure 13. ToF-SIMS spectra (negative ion mode) of Os_3-COOH nanotags. . (a) Full spectrum of ToF-SIMS of Os_3-COOH nanotags. (b) Calculated isotope distribution. (c) Isotope distribution obtained from experiment.

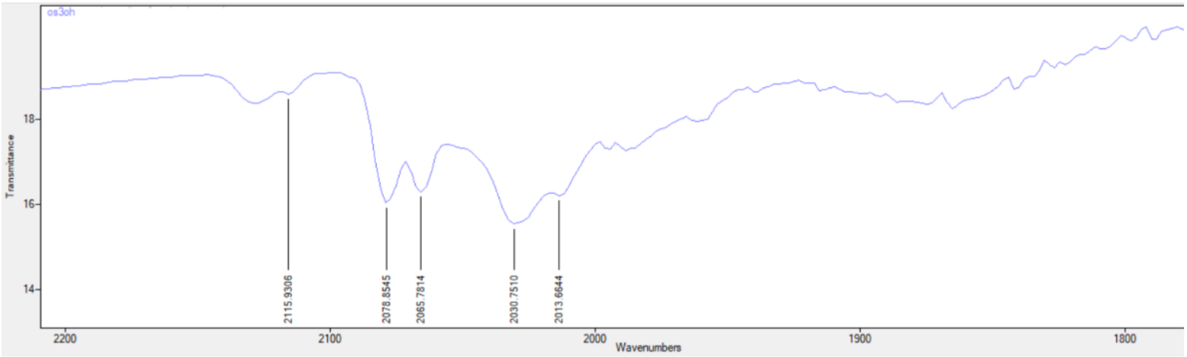

Supplementary Figure 14. IR spectrum of an Os_3-OH nanotag.

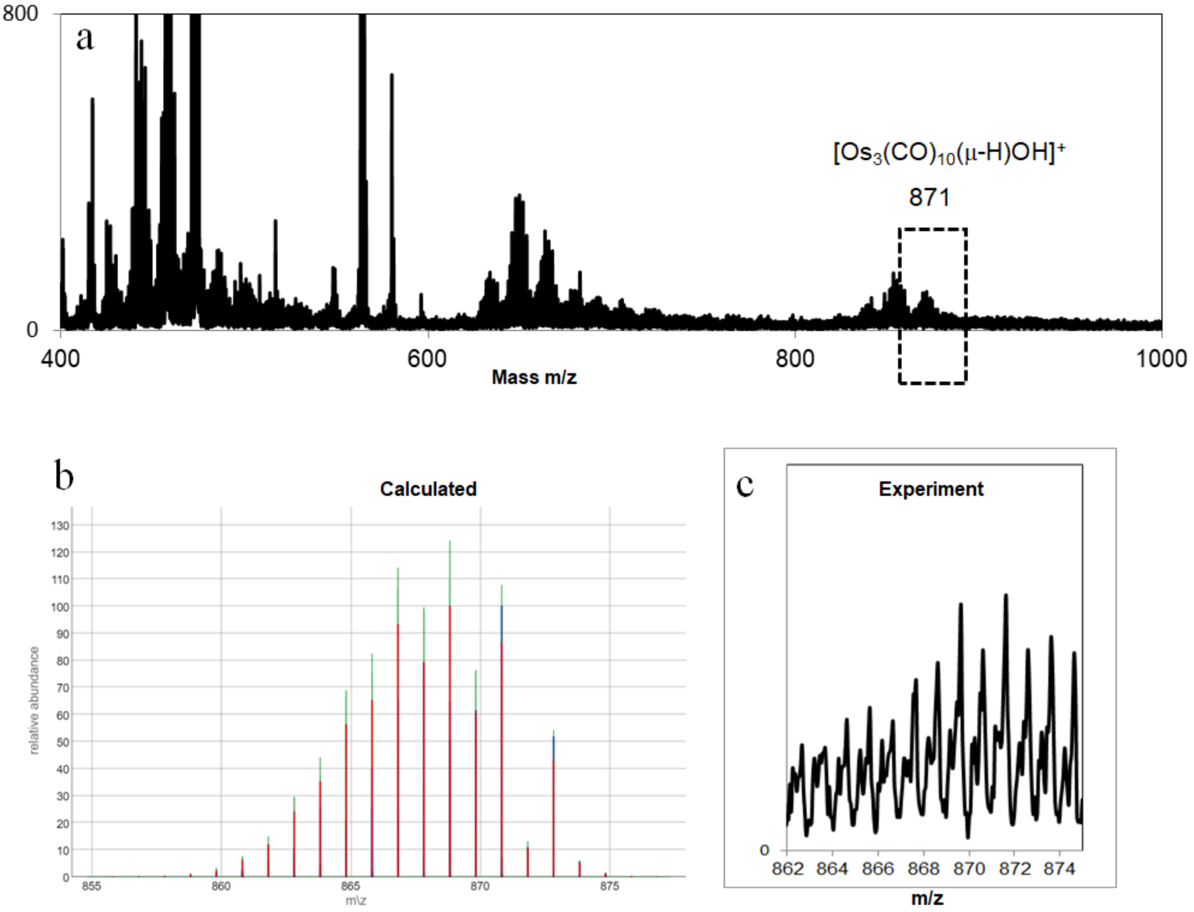

Supplementary Figure 15. ToF-SIMS spectra (negative ion mode) of Os_3-OH nanotags. (a) Full spectrum of ToF-SIMS of Os_3-OH nanotags. (b) Calculated isotope distribution. (c) Isotope distribution obtained from experiment.

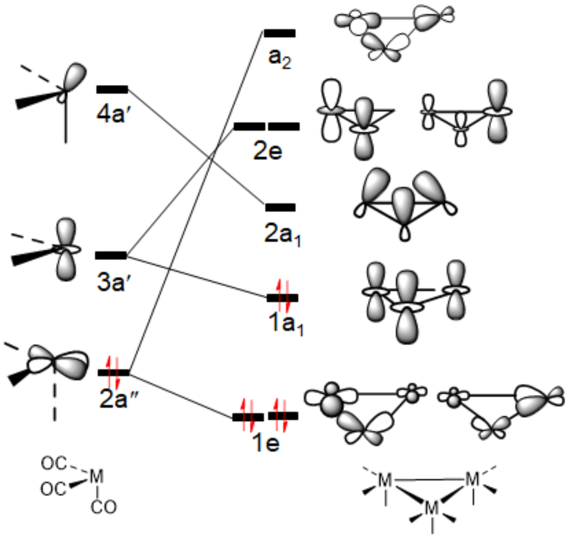

Supplementary Figure 16. Partial MO diagram for $M_3(CO)_{12}$.[2]

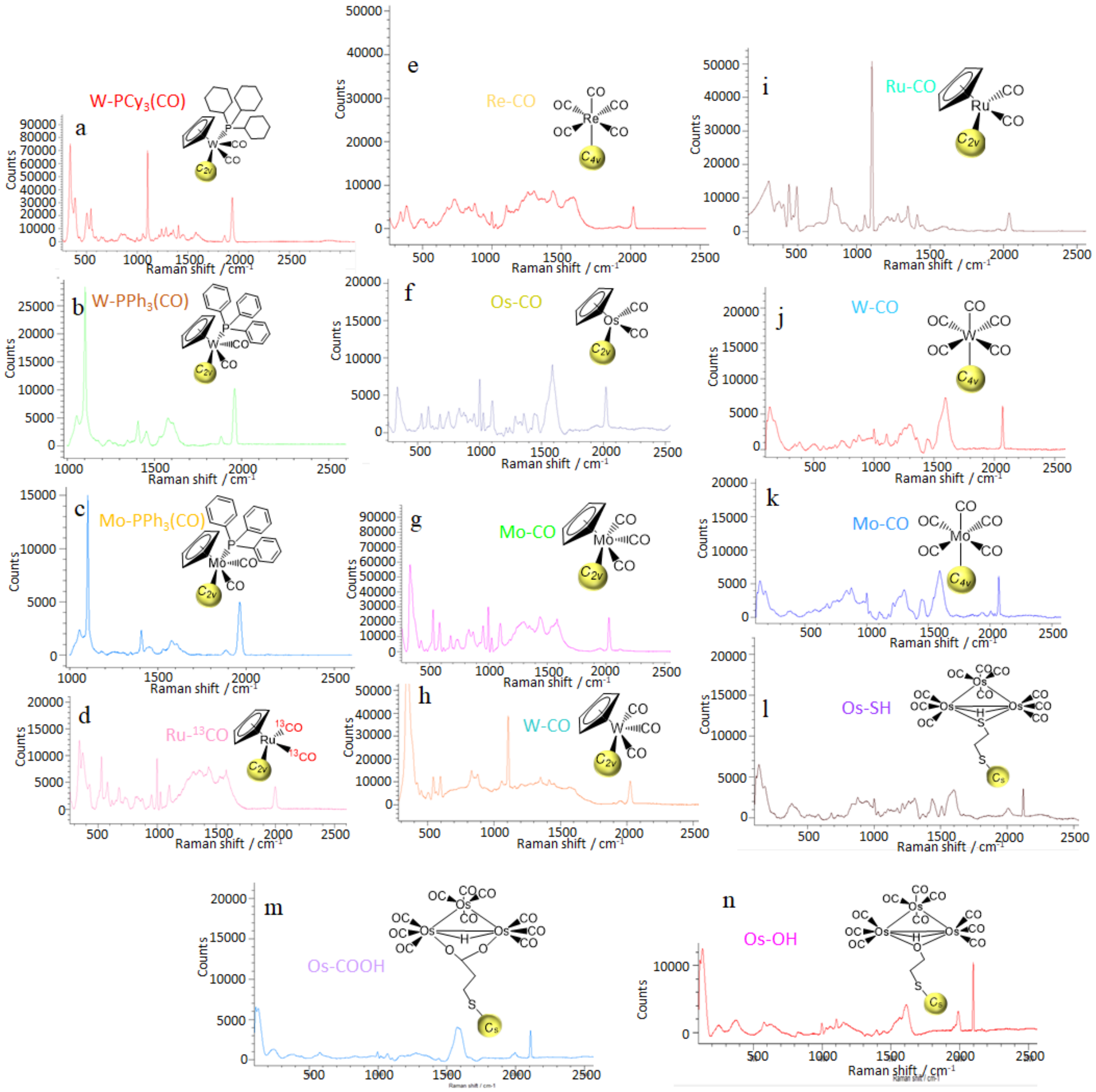

Supplementary Figure 17. Full range Raman spectra for all 14 nanotags. (a) $CpWPCy_3(CO)_2$-Au, (b) $CpWPPh_3(CO)_2$-Au, (c) $CpMoPPh_3(CO)_2$-Au, (d) $CpRu(^{13}CO)_2$-Au, (e)$Re(CO)_5$-Au, (f) $CpOs(CO)_2$-Au, (g) $CpMo(CO)_3$-Au, (h) $CpW(CO)_3$-Au, (i) $CpRu(CO)_2$-Au, (j) $W(CO)_5$-Au, (k) $Mo(CO)_5$-Au, (l) $(Os_3(CO)_{10}(\mu\text{-}S(CH_2)_8S$-Au, (m) $Os_3(CO)_{10}(\mu,\eta^2OOC(CH_2)_{10}S$-Au, (n) $Os_3(CO)_{10}(\mu\text{-}OC(CH_2)_{10}S$-Au.

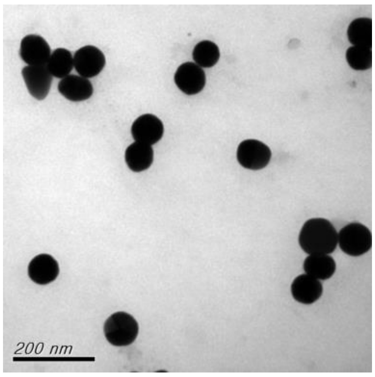

Supplementary Figure 18. TEM images of the nanotags (Os_3-SH nanotag). A representative image of three individual experiments is shown (scale bar: 200 nm).

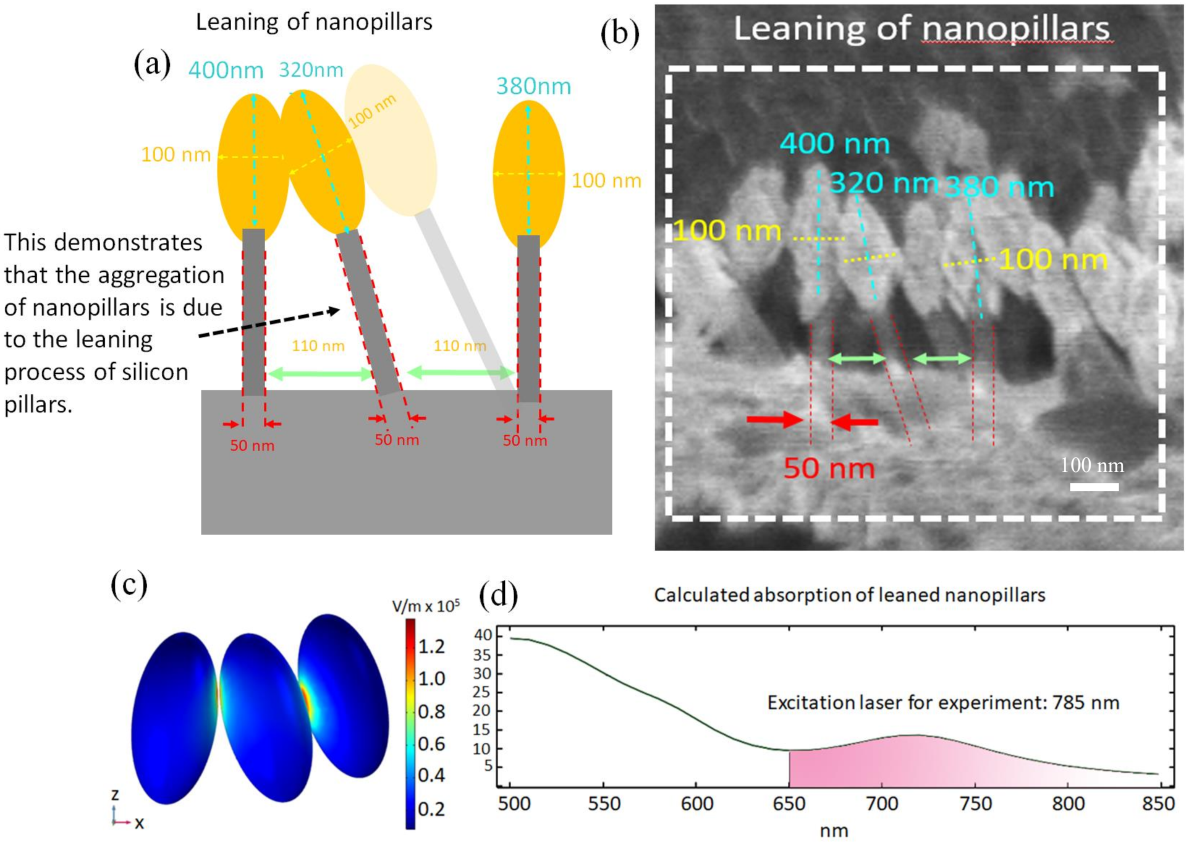

Supplementary Figure 19. Leaned nanopillars analysis. (a) Schematic of leaned nanopillars. (b) SEM image of clustered nanopillars due to the leaning process of silicon pillars. A representative image of three individual experiments is shown (scale bar: 100 nm). (c) COMSOL multiphysics simulation of leaned nanopillars. Strong electronmagnetic field is generated from leaned nanopillars. (d) Calculated absorption spectrum of leaned nanopillars. The calculated resonance optical properties of nanopillar substrates (~ 700 nm) confirm the use of a 785 nm excitation laser source for the measurement. Such a substrate size can provide

3000 sample spots for measurement (with laser spot size ~ 1 μm^2) and requires only 5 μL of sample volume to cover the surface of the substrate for biosensing. Nanopillars are known to form microsized nanopillar clusters by leaning towards one another because of the surface tension among nanopillars during incubation and washing.[3-5]

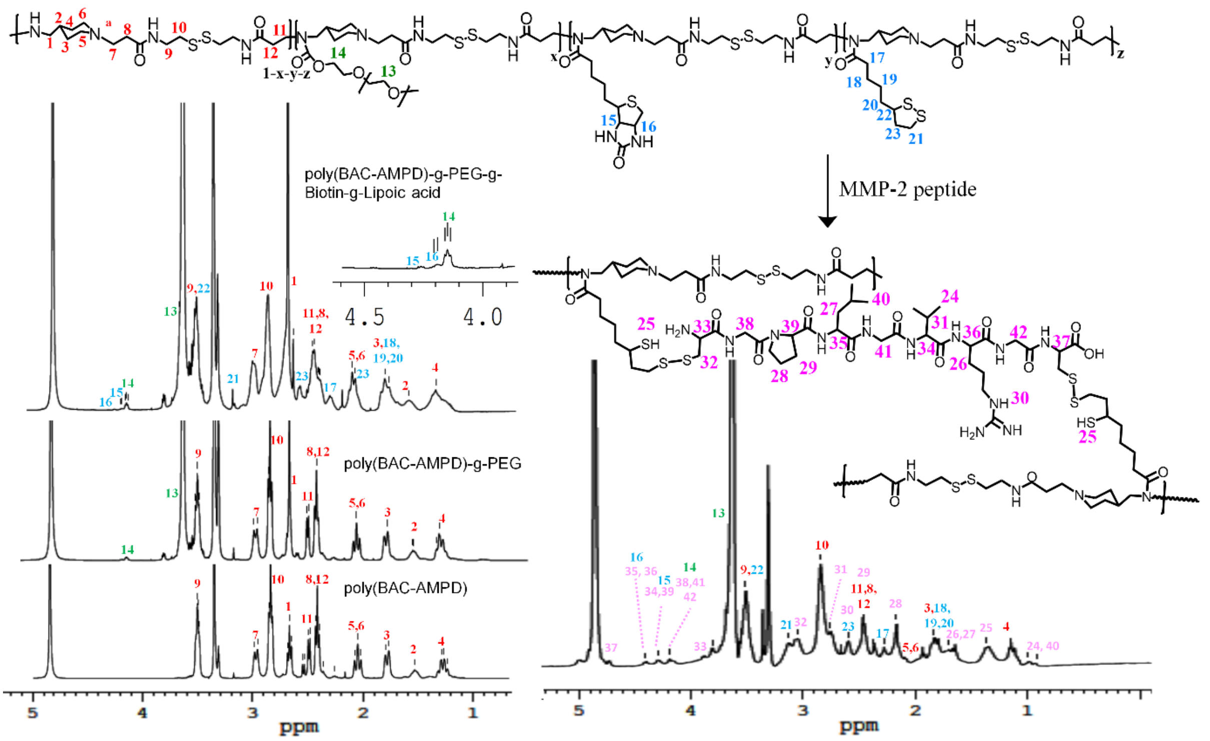

Supplementary Figure 20. NMR spectra of poly(BAC-AMPD)s and after conjugation of biotin and lipoic acid and crosslinking with the MMP-2 peptide. The secondary amines readily react with 4-nitrophenyl carbonate-activated PEG to form PEG-grafted poly(amido amine). The composition of poly(AMPD-BAC)-g-PEG was determined by ^{1}H-NMR measurement from the peak intensity ratio between PEG (4.14 ppm) and AMPD-BAC (1.52 ppm) (Supplementary Fig. 20). The feed molar ratio of PEG to the 2 °amine in poly(BAC-AMPD) was kept at 1:5 to control the amount of PEG grafted and thereby retain some of the secondary amines for further functionalization. The molar ratio of the grafted PEG and BAC-AMPD units was determined to be 1:5 using integrated peak intensity of 4.20 ppm divided by integrated

peak intensity of 1.29 ppm. Biotin and lipoic acid were conjugated to poly(BAC-AMPD)-*g*-PEG via the reaction of the remaining secondary amines in poly(BAC-AMPD) to form amide bonds. The grafting of biotin and lipoic acid was confirmed by the appearance of characteristic peaks, such as peaks (peak intensity ratio between the lipoyl unit (3.1 ppm) and AMPD-BAC (2.9 ppm) in NMR). The molar ratio of the grafted biotin and lipoic acid units was determined to be 1:8:1. The MMP peptide with two sulfhydryl functional groups from the cysteine moiety was used to form a crosslinked gel via lipoic acid. The crosslinking process was confirmed by the disappearance of the absorption band of lipoic acid. The composition of poly(AMPD-BAC)-*g*-PEG-*g*-biotin-*g*-lipoic acid crosslinked MMPs was confirmed by the characteristic peak of the MMP. The free sulfhydryl groups, which are also confirmed by NMR, will be used to immobilize the gel on the Au nanopillar substrate *via* thiol-Au interactions.

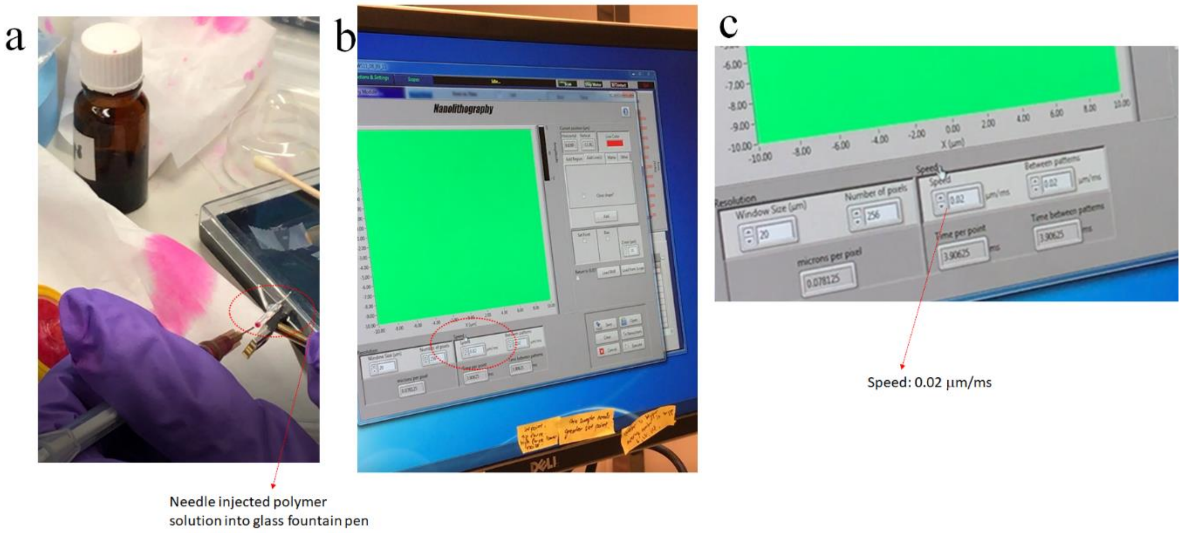

Supplementary Figure 21. Deposition of nanogel using fountain pen. (a) Demonstration of filling the polymer liquid into a fountain pen. (b) Operation platform for fountain pen. (c) Operation platform indicating the drawing was performed at a speed of 0.02 μm/ms.

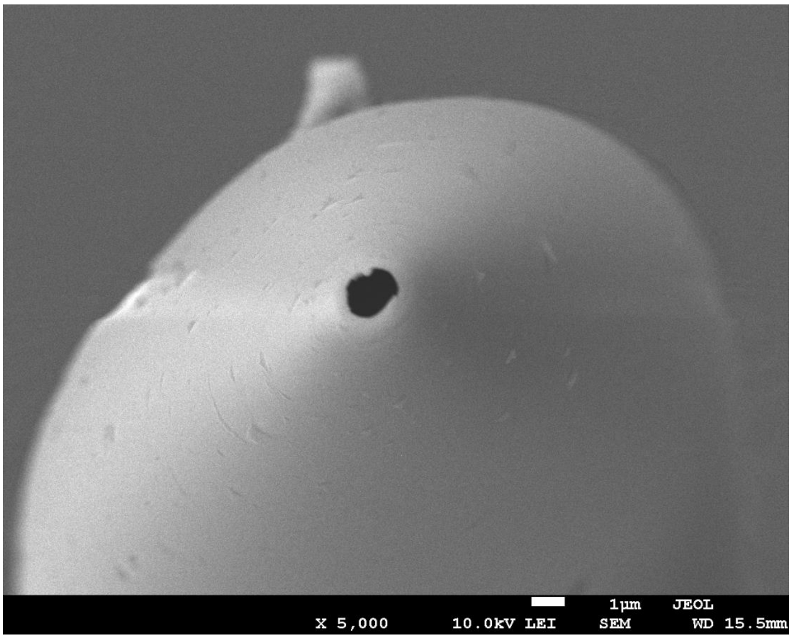

Supplementary Figure 22. SEM image of fountain pen with a small aperture. A representative image of three individual experiments is shown (scale bar: 1 µm).

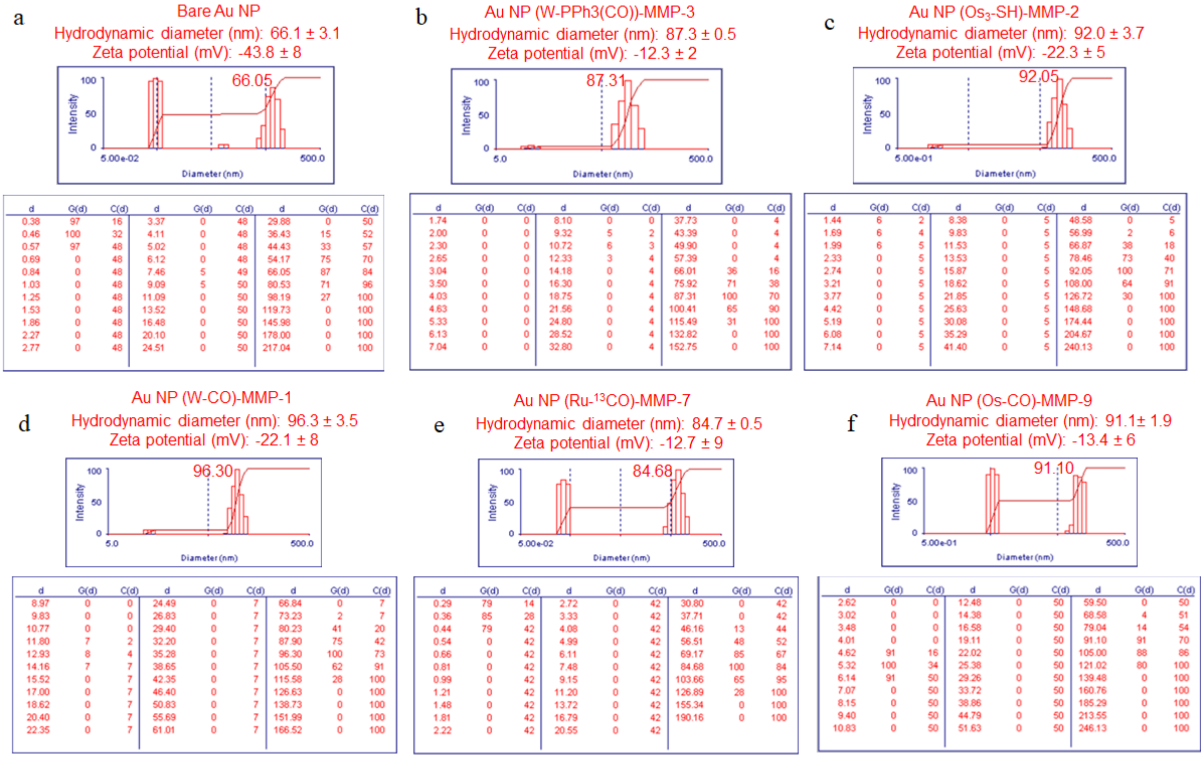

Supplementary Figure 23. Hydrodynamic diameter and zeta potential of nanotags. Dynamic light scattering measurements indicated that the size increased upon conjugation of PEG-MMP peptide, which is commonly observed from PEG.[6] The positive value of the z potential also supported the existence of amine groups of peptides on the surface, which was contrary to the

negative value of sodium citrate gold nanoparticles.[7] (a) Bare Au NP, (b) Au NP (W-PPh_3(CO))-MMP-3, (c) Au NP (Os_3-SH)-MMP-2, (d) Au NP (W-CO)-MMP-1, (e) Au NP (Ru-^{13}CO)-MMP-7, (f) Au NP (Os-CO)-MMP-9.

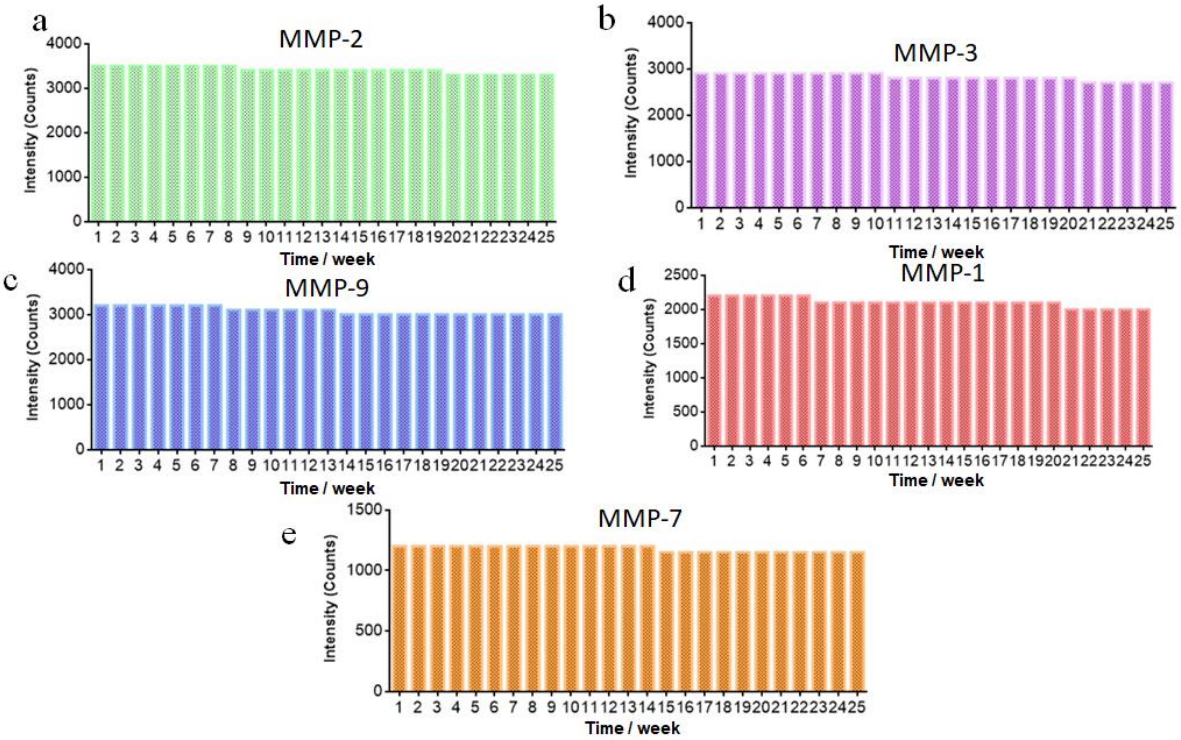

Supplementary Figure 24. Signals of nanotags observed over 6 months. (a) Os-SH-Au-MMP-2-PEG, (b) $CpWPPh_3(CO)_2$-Au-MMP3-PEG, (c) $CpMo(CO)_3$-MMP-9-PEG, (d) $W(CO)_5$-MMP-1-PEG, (e) $CpRu(^{13}CO)_2$-MMP-7-PEG

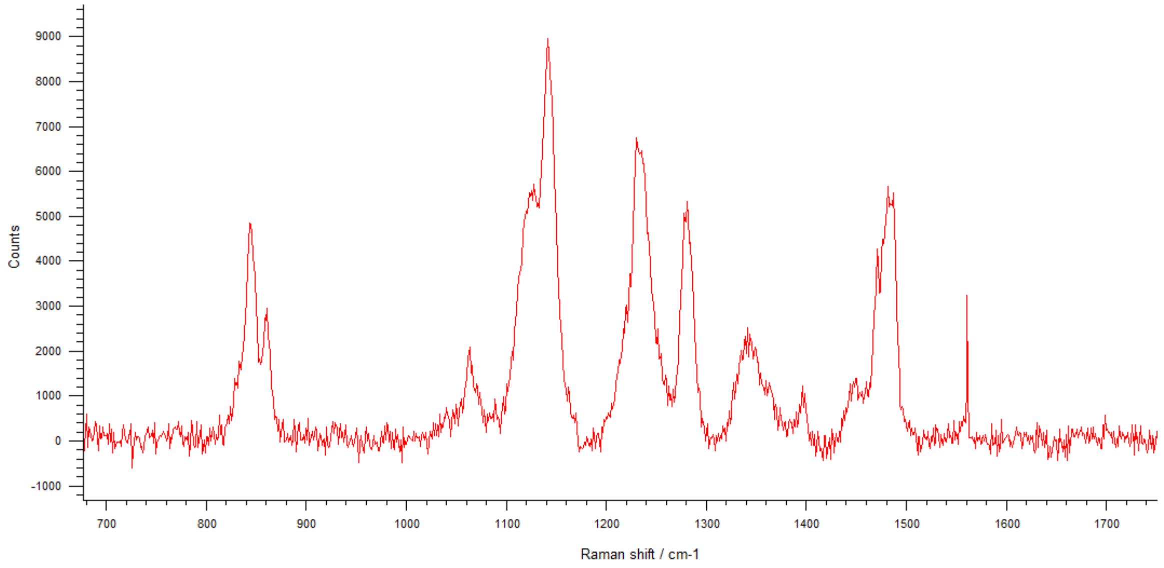

Supplementary Figure 25. PEG-peptide SERS spectrum. The coverage density of peptide-PEG was estimated. Because peptide-PEG has a peak at 1220 cm^{-1}, we are able to use this peak to measure the concentration of PEG from unreacted peptide-PEG molecules left behind in the process, which can be achieved by measuring the supernatant.

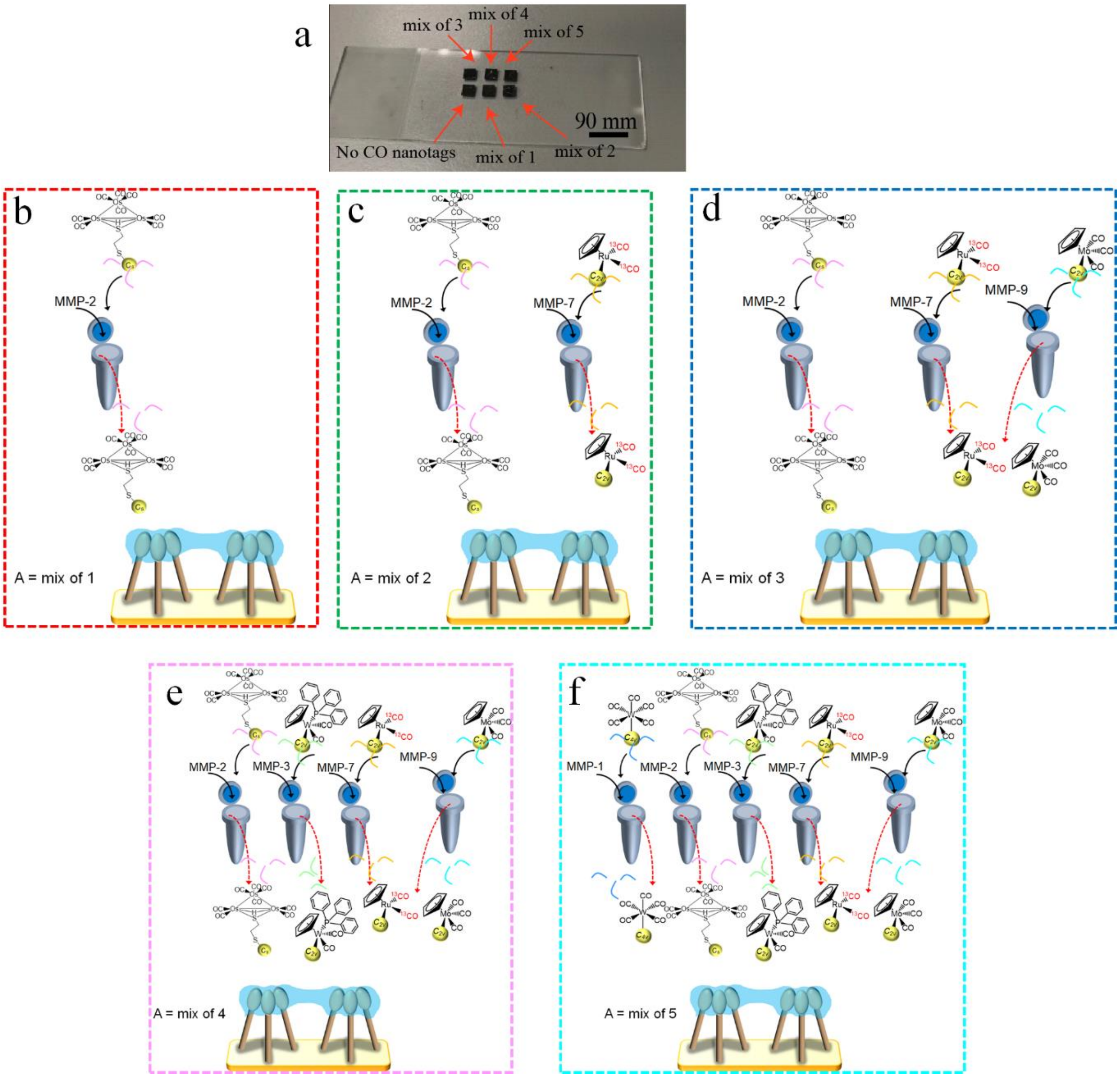

Supplementary Figure 26. Schematic representation of unmixed colocalization of five mixtures of MMPs. (a) Nanogel substrates for multiple colocalized SERS detection, (b) Mixing of single nanotag for MMP-2 detection, (c) Mixing of two nanotags for MMP-2 and MMP-7 detection, (d) Mixing of three nanotags for MMP-2, MMP-7 and MMP-9 detection, (e) Mixing of four nanotags for MMP-2, MMP-3, MMP-7 and MMP-9 detection, (f) Mixing of five nanotags for MMP-1, MMP-2, MMP3, MMP-7 and MMP-9 detection.

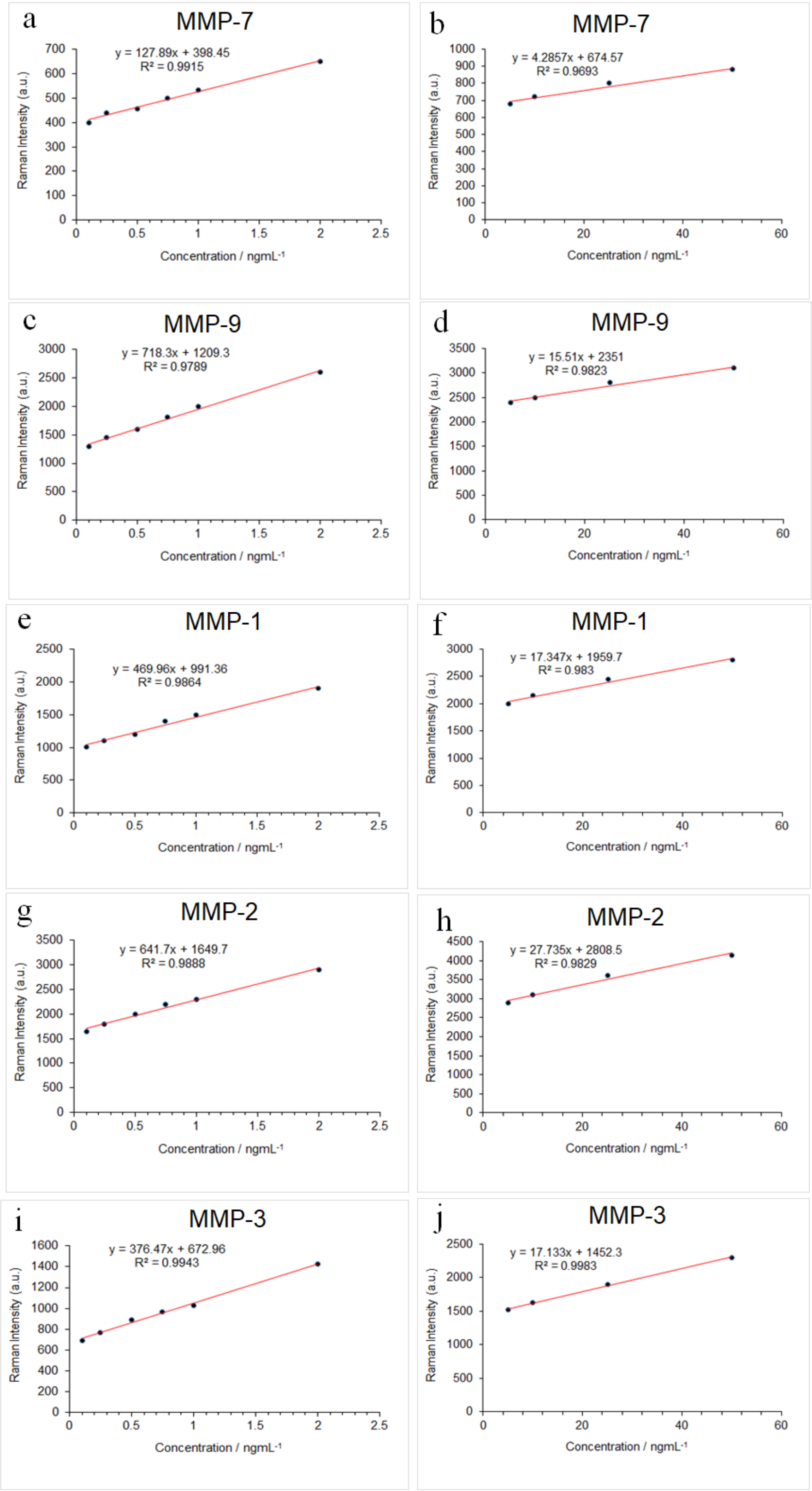

Supplementary Figure 27. The SERS detection for (a-b) MMP-7, (c-d) MMP-9, (e-f) MMP-1, (g-h) MMP-2, (i-j) MMP-3. A good linear relationship between the concentration of MMPs and Raman intensity can be obtained for different detection ranges via segment fit processing.

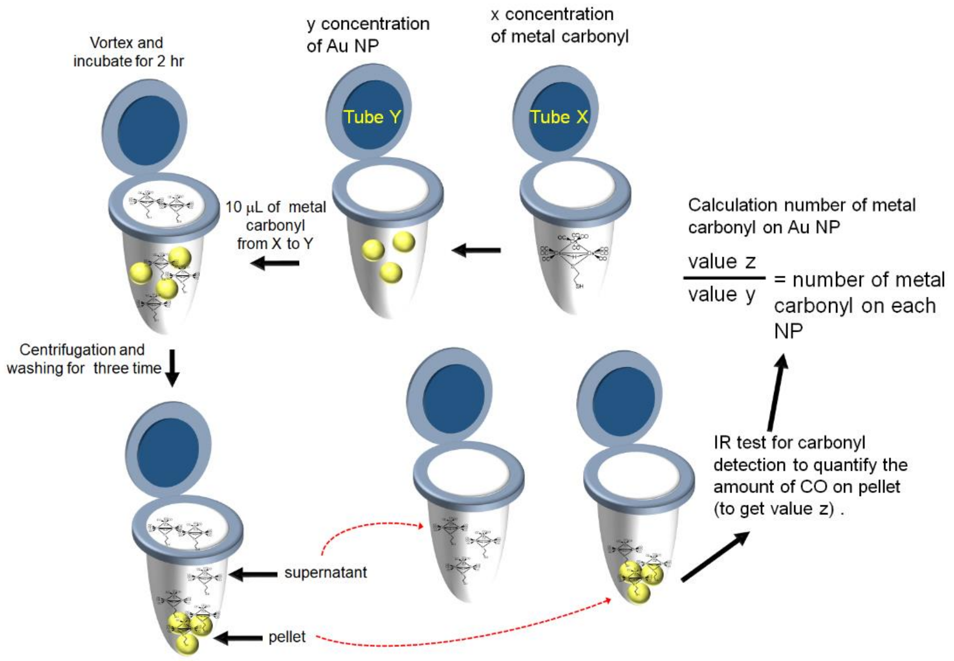

Supplementary Figure 28. Preparation of SERS nanotags. The concentration of metal carbonyl on gold nanoparticles was estimated by IR test.

Supplementary Table 1. The coefficient of variation (CV) for SERS sensors.

	MMP-1	MMP-2	MMP-3	MMP-7	MMP-9
$CV_{chip\text{-}to\text{-}chip}$	6.2%	5.3%	6.4%	6.8%	6.1%
$CV_{batch\text{-}to\text{-}batch\ nanotags}$	9.3%	9.1%	8.3%	8.7%	9.8%

CV: coefficient of variation (standard deviation/average x 100)

Supplementary Table 2. Patient characteristics

Number	Age	Sex	TNM	Stage	EBV-DNA (copies/mL)
1	45	Male	$T_1N_0M_0$	I	1180
2	46	Male	$T_1N_0M_0$	I	200
3	43	Female	$T_1N_0M_0$	I	500
4	45	Female	$T_2N_0M_0$	II	1400
5	62	Male	$T_2N_1M_0$	II	211
6	25	Female	$T_1N_1M_0$	II	331
7	39	Male	$T_2N_0M_0$	II	500
8	35	Female	$T_1N_1M_0$	II	733
9	63	Female	$T_1N_1M_0$	II	285
10	44	Male	$T_2N_0M_0$	II	1250
11	50	Female	$T_3N_1M_0$	III	1100
12	31	Male	$T_3N_2M_0$	III	4500
13	37	Male	$T_3N_2M_0$	III	1320
14	20	Female	$T_2N_2M_0$	III	4210
15	26	Male	$T_3N_2M_0$	III	16400
16	63	Male	$T_3N_0M_0$	III	854
17	53	Male	$T_2N_2M_0$	III	598
18	44	Male	$T_2N_2M_0$	III	1310
19	28	Female	$T_2N_2M_0$	III	5320
20	59	Male	$T_2N_2M_0$	III	100000
21	33	Female	$T_3N_3M_0$	IV	6570
22	52	Male	$T_4N_2M_0$	IV	5810
23	65	Male	$T_4N_2M_0$	IV	24400
24	66	Male	$T_4N_2M_0$	IV	5980
25	40	Male	$T_2N_3M_0$	IV	26400

26	22	Male	$T_4N_3M_0$	IV	238000
27	64	Male	$T_4N_1M_0$	IV	2830000
28	52	Male	$T_4N_1M_0$	IV	867
29	28	Female	$T_4N_1M_0$	IV	16900
30	62	Male	$T_4N_2M_0$	IV	1500

Supplementary Table 3. Concentration of MMPs in clinical blood samples measured by SERS and ELISAs. P: NPC patient. Each value is the mean of triplicate assays.

Subject	MMP-1 (ng/mL)		MMP-2 (ng/mL)		MMP-3 (ng/mL)		MMP-7 (ng/mL)		MMP-9 (ng/mL)	
	SERS	ELISA	SERS	ELISA	SERS	ELISA	SERS	ELISA	SERS	ELISA
P-1	10.6	11.4	322.2	307.7	155.5	169.6	6.0	4.9	10.2	7.4
P-2	4.3	3.9	276.8	298.2	156.3	148.0	5.2	4.3	14.1	13.3
P-3	6.9	6.4	379.6	357.6	33.5	35.0	6.2	5.6	11.2	10.7
P-4	5.0	3.9	542.3	492.4	175.4	181.0	5.9	5.7	11.9	10.4
P-5	14.5	12.8	936.4	1029.6	172.0	182.2	6.1	6.6	15.7	14.5
P-6	9.8	8.8	588.1	540.1	58.8	53.1	7.8	5.8	32.0	27.6
P-7	6.5	6.2	542.3	502.5	319.3	286.5	7.0	6.1	18.3	18.5
P-8	17.3	19.0	450.0	404.5	91.3	85.9	5.3	6.0	13.8	14.7
P-9	23.0	21.2	570.5	523.4	70.5	98.1	6.8	6.9	21.7	20.1
P-10	6.0	5.0	746.3	675.9	155.2	165.5	9.3	9.6	18.6	17.8
P-11	26.0	23.2	310.8	282.2	68.0	65.6	8.0	6.7	13.6	14.3
P-12	25.7	23.7	333.6	352.8	130.6	112.6	9.6	8.5	17.2	17.8
P-13	12.6	13.4	392.3	360.9	70.2	58.1	4.8	5.2	40.8	30.6
P-14	9.0	9.2	1102.3	1039.6	12.2	16.6	6.1	5.8	27.8	28.8
P-15	33.1	31.8	440.5	397.4	44.5	42.5	6.8	6.1	22.2	22.7
P-16	10.1	10.6	1001.1	921.0	210.6	200.5	4.1	3.4	20.1	19.3
P-17	14.9	15.4	440.7	492.7	193.7	171.8	6.0	7.1	48.8	45.2
P-18	22.8	23.8	370.9	394.3	204.4	198.3	9.1	9.8	21.1	19.8
P-19	32.9	31.5	345.1	316.4	75.8	71.4	7.0	6.6	23.8	21.1
P-20	9.8	9.1	820.0	721.4	130.7	112.0	17.5	14.4	19.3	21.7
P-21	46.2	50.3	266.6	289.2	72.2	83.9	10.6	11.5	98.8	87.1
P-22	25.0	22.0	420.6	344.9	96.8	87.9	9.0	8.4	40.1	35.2
P-23	40.1	36.9	360.7	389.3	93.3	88.8	17.8	19.2	32.1	30.0
P-24	33.4	35.0	400.8	423.5	79.9	70.8	8.8	9.5	48.2	51.2
P-25	163.3	151.0	377.8	392.1	67.8	56.5	20.1	18.1	38.1	34.4
P-26	53.1	51.5	380.1	440.1	78.5	63.1	58.1	63.1	82.2	86.3
P-27	28.5	28.8	300.8	325.5	108.6	113.2	8.8	9.2	112.1	104.7
P-28	26.0	24.1	330.3	290.3	74.8	61.5	9.1	8.8	67.3	57.3

P-29	40.5	39.3	300.5	339.3	73.9	60.5	6.6	6.5	35.2	30.2
P-30	49.3	45.8	288.4	292.0	90.5	97.6	6.1	5.9	58.6	60.1

Supplementary Table 4. The *P* values for the correlation between the clinical pathological parameters and MMPs level detected by the SERS sensor.

MMP	Combination	*P* value
MMP 1	Early vs. advanced stage	0.00043
	T1 vs. T2	0.83
	T1 vs. T3	0.041
	T1 vs. T4	0.00067
	T2 vs. T3	0.15
	T2 vs. T4	0.0085
	T3 vs. T4	0.12
	N0 vs. N1	0.0031
	N0 vs. N2	0.00048
	N0 vs. N3	0.017
	N1 vs. N2	0.88
	N1 vs. N3	0.019
	N2 vs. N3	0.0088
MMP 2	Early vs. advanced stage	0.11
	T1 vs. T2	0.25
	T1 vs. T3	0.7
	T1 vs. T4	0.34
	T2 vs. T3	0.12
	T2 vs. T4	0.0087
	T3 vs. T4	0.57
	N0 vs. N1	0.69
	N0 vs. N2	0.7
	N0 vs. N3	0.25
	N1 vs. N2	0.85

	N1 vs. N3	0.5
	N2 vs. N3	0.29
MMP 3	Early vs. advanced stage	0.13
	T1 vs. T2	0.18
	T1 vs. T3	0.9
	T1 vs. T4	0.75
	T2 vs. T3	0.26
	T2 vs. T4	0.12
	T3 vs. T4	0.34
	N0 vs. N1	0.054
	N0 vs. N2	0.056
	N0 vs. N3	0.12
	N1 vs. N2	0.42
	N1 vs. N3	0.63
	N2 vs. N3	0.23
MMP 7	Early vs. advanced stage	0.043
	T1 vs. T2	0.13
	T1 vs. T3	0.52
	T1 vs. T4	0.017
	T2 vs. T3	0.7
	T2 vs. T4	0.37
	T3 vs. T4	0.33
	N0 vs. N1	0.19
	N0 vs. N2	0.099
	N0 vs. N3	0.017
	N1 vs. N2	0.54
	N1 vs. N3	0.012

	N2 vs. N3	0.025
MMP 9	Early vs. advanced stage	0.00016
	T1 vs. T2	0.15
	T1 vs. T3	0.18
	T1 vs. T4	0.00067
	T2 vs. T3	0.79
	T2 vs. T4	0.0014
	T3 vs. T4	0.081
	N0 vs. N1	0.054
	N0 vs. N2	0.00048
	N0 vs. N3	0.017
	N1 vs. N2	0.57
	N1 vs. N3	0.13
	N2 vs. N3	0.07

Supplementary Table 5. Comparison between the SERS detection method and other analytical methods.

Category	SERS detection<br>(This work)	Colorimetric assay<br>(*Nat. Nanotech*. 2019, 14, 883)	Potentiometric detection<br>*(Nat. Common. 2017, 8, 264)*
Biomarkers	MMP-1, MMP-2, MMP-3,<br>MMP-7, MMP-9	MMP-9	MMP-8
Description	Detection of five biomarkers by measuring the changes in the SERS signals of nanotags	Detection of single biomarker (MMP-9) by measuring the changes in the colour of solution due to gold nanoclusters.	Detection of single biomarker (MMP-8) by measuring the changes in potential.
Equipment for measurement	Raman spectrometer	UV-vis spectrometer	Potentiometric electronic taste sensing system
Sample and sample volume	Plasma (5 μL)	Urine (25 μL)	Saliva (50 μL)
Time for detection	20 min	1 h	<10 min<br>No exact measurement time indicated.
LOD<br>(R^2 value)	0.07 ng/mL for MMP-1<br>($R^2 = 0.99$)<br>, 0.083 ng/mL for MMP-2<br>($R^2 = 0.99$),<br>0.1 ng/mL for MMP-3<br>($R^2 = 0.98$),<br>0.14 ng/mL for MMP-7<br>($R^2 = 0.98$),<br>0.11 ng/mL for MMP-9<br>($R^2 = 0.98$)	2.7 pmol in 25 μL<br>($R^2 = 0.9996$)<br>*in supporting information: MMP9 stock (Merck PF140 lot#2872521, 0.1 mg mL-1 ~ 1500 nM)*	10 nM<br>(-)

Supplementary Table 6. The primers used for EBV-DNA detection in PCR.

Name	Sequence
EBV-F	TGCCAAAGAGCCAGATCTAAGG
EBV-R	AAAGTGTCAGATTTTGGGTCCAA
EBV-P	FAM-CAGCCCCAAAGCGGGTGCAGTAAC-BHQ1

References

1. Sharpe, C. H. a. A. G. *Inorganic Chemistry (5th Edition)*. (Pearson Education, Press, Harlow, 2018).
2. Schilling, B. E. R., Hoffmann, R. M_3L_9(ligand) complexes. *J. Am. Chem. Soc.* **101**, 3456-3467 (1979).
3. Gong, T., Hong, Z.-Y., Chen, C.-H., Tsai, C.-Y., Liao, L.-D., Kong, K. V. Optical Interference-Free Surface-Enhanced Raman Scattering CO-Nanotags for Logical Multiplex Detection of Vascular Disease-Related Biomarkers. *ACS Nano* **11**, 3365-3375 (2017).
4. Kim, A., Ou, F. S., Ohlberg, D. A. A., Hu, M., Williams, R. S., Li, Z. Study of Molecular Trapping Inside Gold Nanofinger Arrays on Surface-Enhanced Raman Substrates. *J. Am. Chem. Soc.* **133**, 8234-8239 (2011).
5. Yang, J., *et al.* Surface-Enhanced Raman Spectroscopy Based Quantitative Bioassay on Aptamer-Functionalized Nanopillars Using Large-Area Raman Mapping. *ACS Nano* **7**, 5350-5359 (2013).
6. Schollbach, M., Zhang, F., Roosen-Runge, F., Skoda, M. W. A., Jacobs, R. M. J., Schreiber, F. Gold nanoparticles decorated with oligo(ethylene glycol) thiols: Surface charges and interactions with proteins in solution. *J. Colloid Interface Sci.* **426**, 31-38 (2014).
7. Shinohara, S., Eom, N., Teh, E. J., Tamada, K., Parsons, D., Craig, V. S. J. The Role of Citric Acid in the Stabilization of Nanoparticles and Colloidal Particles in the Environment: Measurement of Surface Forces between Hafnium Oxide Surfaces in the Presence of Citric Acid. *Langmuir* **34**, 2595-2605 (2018).
