# Supplementary material for: Geometrically encoded SERS nanobarcodes for the logical detection of nasopharyngeal carcinoma-related progression biomarkers
Source: Nat Commun. 2021 Jun 8;12:3430. doi: 10.1038/s41467-021-23789-3 (PMC8173014; doi:10.1038/s41467-021-23789-3)
Supplement: Supplementary file 2 — Reporting Summary [file 41467_2021_23789_MOESM2_ESM.pdf]

nature research

Corresponding author(s): Kien Voon Kong

Last updated by author(s): April 30, 2021

# Reporting Summary

Nature Research wishes to improve the reproducibility of the work that we publish. This form provides structure for consistency and transparency in reporting. For further information on Nature Research policies, see our Editorial Policies and the Editorial Policy Checklist.

## Statistics

For all statistical analyses, confirm that the following items are present in the figure legend, table legend, main text, or Methods section.

| n/a | Confirmed | |
|---|---|---|
| | ☒ | The exact sample size ($n$) for each experimental group/condition, given as a discrete number and unit of measurement |
| | ☒ | A statement on whether measurements were taken from distinct samples or whether the same sample was measured repeatedly |
| | ☒ | The statistical test(s) used AND whether they are one- or two-sided<br>*Only common tests should be described solely by name; describe more complex techniques in the Methods section.* |
| ☒ | | A description of all covariates tested |
| | ☒ | A description of any assumptions or corrections, such as tests of normality and adjustment for multiple comparisons |
| | ☒ | A full description of the statistical parameters including central tendency (e.g. means) or other basic estimates (e.g. regression coefficient) AND variation (e.g. standard deviation) or associated estimates of uncertainty (e.g. confidence intervals) |
| | ☒ | For null hypothesis testing, the test statistic (e.g. $F$, $t$, $r$) with confidence intervals, effect sizes, degrees of freedom and $P$ value noted<br>*Give $P$ values as exact values whenever suitable.* |
| ☒ | | For Bayesian analysis, information on the choice of priors and Markov chain Monte Carlo settings |
| ☒ | | For hierarchical and complex designs, identification of the appropriate level for tests and full reporting of outcomes |
| | ☒ | Estimates of effect sizes (e.g. Cohen's $d$, Pearson's $r$), indicating how they were calculated |

*Our web collection on statistics for biologists contains articles on many of the points above.*

## Software and code

Policy information about availability of computer code

| | |
|---|---|
| Data collection | Wire 4.3 software was used to record the SERS spectra.<br>SoftMax Pro7 software from Absorbance Reader CMax Plus was used to record the Elisa results.<br>COMSOL Multiphysics 5.3 was used to calculate electromagnetic fields. |
| Data analysis | Curve fitting analysis was performed by Wire 4.3 software.<br>Correlation analysis was performed using the Sangerbox tools,an online platform for data analysis (http://www.sangerbox.com/tool). |

For manuscripts utilizing custom algorithms or software that are central to the research but not yet described in published literature, software must be made available to editors and reviewers. We strongly encourage code deposition in a community repository (e.g. GitHub). See the Nature Research guidelines for submitting code & software for further information.

## Data

Policy information about availability of data

All manuscripts must include a data availability statement. This statement should provide the following information, where applicable:

- Accession codes, unique identifiers, or web links for publicly available datasets
- A list of figures that have associated raw data
- A description of any restrictions on data availability

Data are available within the article and supplementary files. The source data underlying Fig. 2c, 2d, 2e, 2f, 5d, 5f, 6a and 6b are provided as a Source Data file. All other data that support the findings of the study are available from the corresponding author upon reasonable request.

# Field-specific reporting

Please select the one below that is the best fit for your research. If you are not sure, read the appropriate sections before making your selection.

☒ Life sciences ☐ Behavioural & social sciences ☐ Ecological, evolutionary & environmental sciences

For a reference copy of the document with all sections, see nature.com/documents/nr-reporting-summary-flat.pdf

# Life sciences study design

All studies must disclose on these points even when the disclosure is negative.

| | |
|---|---|
| Sample size | A total of 30 blood plasma samples were collected in this study, including 3 samples from NPC patients with stage Ⅰ, 7 samples from NPC patients with stage Ⅱ, 10 samples from NPC patients with stage Ⅲ and 10 samples from NPC patients with stage Ⅳ determined by standard histopathological diagnosis. In clinical practice, NPC patients with stage I are extremely rare since early NPC is relatively asymptomatic. Over 80% of the patients with NPC present with locally advanced disease or distant metastasis at diagnosis. Thus, the number of samples from patients with early stage NPC involved in this study is reasonable. |
| Data exclusions | No sample was excluded from the present analysis. All samples were included for statistical analysis. The level of statistical significance or correlation is indicated in the Figure 7, Figure 8 and Figure 9. |
| Replication | All experimental were reliably reproducible. The data are based on at least three independent experiments with similar results. The number of repeats is given in the legend of Figures. |
| Randomization | We did not use randomization to assign the participants to experimental groups. The samples of experimental each group were provided by Fujian Cancer Hospital. |
| Blinding | All patient sample experiments were done in a blinded manner. All patient samples had been de-identified by a research assistant who is not involved in the study. The results were only revealed after the completion of SERS experiments. |

# Reporting for specific materials, systems and methods

We require information from authors about some types of materials, experimental systems and methods used in many studies. Here, indicate whether each material, system or method listed is relevant to your study. If you are not sure if a list item applies to your research, read the appropriate section before selecting a response.

## Materials & experimental systems

| n/a | Involved in the study | |
|---|---|---|
| ☒ | ☐ | Antibodies |
| ☒ | ☐ | Eukaryotic cell lines |
| ☒ | ☐ | Palaeontology and archaeology |
| ☒ | ☐ | Animals and other organisms |
| ☐ | ☒ | Human research participants |
| ☒ | ☐ | Clinical data |
| ☒ | ☐ | Dual use research of concern |

## Methods

| n/a | Involved in the study | |
|---|---|---|
| ☒ | ☐ | ChIP-seq |
| ☒ | ☐ | Flow cytometry |
| ☒ | ☐ | MRI-based neuroimaging |

## Human research participants

Policy information about studies involving human research participants

| | |
|---|---|
| Population characteristics | The participants were aged 20 to 66 years. The plasma samples tested were a total of 30. The samples tested consisted of 20 males and 10 females. |
| Recruitment | The blood plasma samples were provided by Fujian Cancer Hospital. |
| Ethics oversight | The study was approved by the Ethics Committee of Fujian Cancer Hospital (SQ2018-010-01). |

Note that full information on the approval of the study protocol must also be provided in the manuscript.
